# Workforce guidelines, burnout, work engagement and the intention to leave: A cross-sectional study among Dutch emergency physicians

**DOI:** 10.1371/journal.pone.0337882

**Published:** 2026-10-05

**Authors:** Annemieke E. Boendermaker, Gideon H. P. Latten, Jelle T. Prins, Kiki M. J. M. H. Lombarts, Paul L. P. Brand

**Affiliations:** 1 Emergency Department, DSEP Section Governance and Wellness, Frisius Medical Center, Heerenveen and Leeuwarden, The Netherlands; 2 Emergency Department, DSEP Scientific Research Section, Maastricht University Hospital, Maastricht, The Netherlands; 3 Department of Clinical Education, Universitair Medisch Centrum Groningen, Groningen, The Netherlands; 4 Department of Professional Performance and Compassionate Care, Medical psychology, Amsterdam Medical Centre, Amsterdam, The Netherlands; 5 Department of Clinical Education, Universitair Medisch Centrum Groningen, Groningen, The Netherlands; Universtiy of Düsseldorf, GERMANY

## Abstract

**Background and importance:**

Emergency medicine physicians (EMPs) work in a dynamic and challenging environment, with high risk of burnout. The prevalences of work engagement, burnout and the intention to leave among Dutch EMP have not been studied before. This study aims to evaluate the adherence to the Dutch Society of Emergency Physicians (DSEP/NVSHA) workforce recommendations, and the association between these recommendations and signs of burnout, work engagement and the intention to leave the profession.

**Methods:**

Cross-sectional online survey based study among all Dutch EMPs in 2019. Two main outcomes, the prevalences of burnout and work engagement, were assessed using the Dutch version of the Maslach Burnout Inventory and the Utrecht Work Engagement Scale. A third main outcome, the intention to leave, was assessed by three self-developed questions. The correlations between the adherence to twelve workforce recommendations and these three main outcomes were assessed using SPSS.

**Main results:**

295 EMPs participated (response rate 56%, 68% female, median age 39 years). Fifty-five respondents (18.5%) met the criteria for burnout. On work engagement, 29 (16.6%) respondents scored low to extremely low (i.e., the 25^th^ groups’ percentile) and 97 (32.9%) respondents scored high to extremely high (i.e., the groups’ 75^th^ percentile). Intention to leave the profession was high with 22–29%. Higher adherence to the workforce recommendations was significantly associated with all three main outcomes, as were five out of twelve separate recommendations.

**Conclusions:**

To ensure a sustainable future in which EMPs are protected from burnout symptoms, supported in their work, and where disproportionate job turnover is minimized, it is crucial these results are met with decisive action. Hospital management must address the unique challenges and risks providing excellent 24/7 emergency care presents. Workforce guidelines are designed to proactively create a more sustainable work environment. This study underscores the necessity of its implementation and creates a foundation for future research.

## Introduction

Emergency medicine physicians (EMPs) work on a 24/7 schedule with unpredictable caseloads and fast-paced decision-making with high impact and emotional strain. To maintain health, work engagement and a fulfilling career, it is important to invest in a sustainable work environment, aimed at preventing burnout and improving career longevity. In 2015, the Dutch Society of Emergency Physicians (DSEP/NVSHA) published its workforce guideline for durable employability and scheduling, stipulating a set of core recommendations and conditions to aspire to [1]. These recommendations are based upon extensive research and similar guidelines from the UK, USA and Australasia [2–4]. Nevertheless, international literature on the effects of adherence to workforce recommendations is lacking and the prevalence of burnout among Dutch EMPs is unknown.

Burnout is recognised worldwide as a syndrome related to a person’s profession. It consists of three domains, which are separately measured: emotional exhaustion, depersonalization and a sense of diminished personal accomplishment [5–8]. Burnout in healthcare providers is associated with diminished mental and physical health, but also with a reduced quality of work, a rise in patient related safety incidents, an increase in the intention to leave the profession and a lasting incapacity for work [9–11].

The opposite of burnout is considered to be work engagement; a positive, fulfilling, work-related state of mind in its own right that is characterised by three psychometrically distinct aspects: vigor, dedication, and absorption [12–14]. Where burnout is mainly predicted by job demands and a lack of job resources, work engagement is exclusively predicted by available job resources [11–14]. Work engagement fosters the intention to remain in the profession through a motivational process, stimulates extra-role behaviour, enhances overall wellbeing, and protects against patient related safety incidents [11–14].

The core recommendations for Dutch EMPs outlined in the workforce guideline address factors presumed to influence burnout and work engagement, such as scheduling and contract conditions. However, these recommendations have not yet been studied as a set. In this cross-sectional study, we aim to estimate the levels of burnout symptoms and work engagement experienced by Dutch EMPs prior to the COVID-19 pandemic, assess self-reported adherence to the different workforce recommendations and examine the association between compliance with these recommendations and the wellness parameters of burnout, work engagement, and intention to leave.

## Methods

### Design and setting

This cross-sectional survey-based study is a first wave of a larger cohort study and was conducted among all 526 Dutch EMPs registered with DSEP at the time. They represent more than 95% of all Dutch EMPs. In the Netherlands EMPs work primarily in the emergency department. From May 1^st^ to June 30^th^ 2019, all were invited via personal e-mail through DSEP to complete an online questionnaire developed by a multidisciplinary research team of EMPs, psychologists, epidemiologists, medical education researchers and human resource management specialists. The questionnaire included internationally validated questions to assess burnout, work engagement and intention to leave the profession, and demographic characteristics, and work situation (S1 Table). After two weeks, non-responders received a reminder to complete the questionnaire.

The study was approved by a regional Dutch ethics review board (RTPO-1046). All participants provided informed consent by checking the consent box at the start of the questionnaire. To further ensure data and privacy safety demographics were separated from the main dataset. Only the two main researchers had the key to link both data sets for analysis purposes.

### Variables and definitions

Demographics – Respondents were asked to provide data on age, gender, relationship status and family composition, year of graduation from medical school, and year of completion of postgraduate EMP training.

Burnout – The Utrecht Burnout Scale (UBOS), the official validated Dutch adaptation of the Maslach Burnout Inventory, was used [15]. Respondents reported their agreement with statements on the three separate domains of burnout – emotional exhaustion (5 items), depersonalization (5 items) and sense of diminished personal accomplishment (7 items) – using a 7-point Likert scale, ranging from “never” (1) to “always” (7). To determine a threshold for ‘burn out’, we used the Brenninkmeijer EEplus1 definition, which requires the presence of emotional exhaustion and either high depersonalization or low personal accomplishment [16]. When emotional exhaustion, high depersonalization and low personal accomplishment were all present, respondents fulfilled the definition of ‘burnout syndrome’ and were also considered to have significant burn out symptomatology [17]. In line with the UBOS manual, the 75^th^ percentile of the distribution of the three composite variables was used as a cut-off for burnout. The scales were compared to the norm reference values of Dutch healthcare personnel presented in the UBOS manual [15]. To clarify its interpretation the UBOS score only indicates a burnout tendency or symptomatology cluster and does not constitute a formal diagnosis of burnout.

Work engagement – The 3-item version of the Utrecht Work Engagement Scale (UWES), which assesses vigor, dedication and absorption, was used [18]. Each item was scored on a 5-point Likert scale from “never” (1) to “always” (5). Overall work engagement was defined and analysed as a composite variable of all three items and compared to the norm reference values provided by the UWES manual of work engagement scores of Dutch healthcare professionals [19].

Work conditions – The work situation was assessed with a model derived from the Dutch Workforce Guideline for Durable Employability and Scheduling [1]. This workforce guideline offers 12 recommendations for working conditions, which were translated to dichotomous questions regarding EMPs’ working conditions. These included scheduled backward rotation, fixed cycle in rotation scheduling, fixed shift off, break time during shift, weekends per month, nightshift to team size, shift duration, contract size, additional scheduled time, presence of ED residents, team size to ED size, and contract type.

Intention to leave the profession [20,21] – Having ever considered to leave emergency medicine was assessed using a 4-point Likert scale, ranging from never (1) to multiple times (4). EMPs were asked, yes or no, whether, given the choice, they would choose the same career again ‘knowing what they know now’, and whether they saw themselves working as an EMP in 10 years’ time?” The choice for a 10-year time horizon was based on the expected young age of the respondent group, given the relative novelty of EMP as a profession in the Netherlands. The answers ‘no’ and ‘often/ multiple times’ were considered poor outcomes.

### Analysis and statistics

To assess the sample’s representativeness of the EMP population, we compared respondents’ gender, age and hospital type to those of all registered EMP members of the DSEP (S2 Table). Descriptive statistics were used to describe the prevalence of the three outcome measures and adherence to the 12 workforce recommendations. We assessed differences in demographic and working conditions using variables between respondents with or without a positive value on the burnout composite score, work engagement and intention to leave using chi-square tests, Fisher exact tests or Mann-Whitney-U tests, as appropriate. Two sided p values <0.05 were considered statistically significant. Cronbach’s alpha was used to measure the internal consistency of construct variables. To assess correlation between the three main outcomes and the workforce recommendations, we computed Spearman rank (r_s_), rank biserial (r_rb_)and phi correlation (*r*_*φ*_) coefficients. Binomial and ordinal logistic regressions were conducted to determine the effects of burnout and work engagement on the likelihood of the intention to leave.

If a respondent wanted to move forward with the online questionnaire all previous questions had to have been answered. So missing items were extremely rare. The entire questionnaire consisted of multiple preset research tools. If there was one item missing within a construct variable single imputation was used and sensitivity analysis was done to confirm robustness. If the entire preset, such as the UBOS or the work conditions, was missing the case was excluded for subsequent analyses. All analyses were performed using IBM SPSS Statistics for Windows, version 27 (IBM Corp., Armonk, N.Y., USA).

## Results

### Demographics

In total, 295 (56%) EMPs completed the questionnaire, of which 200 (67.8%) were female. Median age was 39 (IQR 35–44) years and respondents had been working as an EMP for a median of 6 (IQR 3–10) years. Age, sex and hospital type were comparable between respondents and the entire cohort of DSEP members (S2 Table).

### Burnout symptoms and work engagement

The internal consistency of the construct variables were excellent, with Cronbach’s alpha for emotional exhaustion α = .91, for depersonalization α = .82, for personal accomplishment α = .81 and α = 0.70 for work engagement. Burnout, defined as emotional exhaustion plus high depersonalization and/or low personal accomplishment, was observed in 55 (18.6%) respondents (Table 1). High emotional exhaustion was present in 27.1%, and low personal accomplishment in 21.6%. Consistent with the UBOS manual, depersonalisation was reported as a male score at 25.0% and female score of 28.0%.

**Table 1:** Demographics and burnout. Numbers are median (IQR) or n (%). P value for categorical variables by X square test, and for comparison of means by independent samples t-test.

Demographics	Total (n = 295)	No burnout (n = 240)	Burnout (n = 55)	p
Sex – female	200 (67.8)	164 (68.3)	36 (65.5)	0.75
Sex – male	95 (32.2)	76 (31.6)	19 (34.5)	
Age – years	39 (35-44)	40 (35-43)	41 (36-45)	0.22
Working as EMP– years	6 (3-10)	6 (3-10)	6 (3-9)	0.98
With partner	247 (83.7)	209 (87.1)	38 (69.1)	0.002
Without partner	48 (16.3)	31 (12.9)	17 (30.9)	
With children	196 (66.4)	159 (66.3)	37 (67.3)	0.89
Without children	99 (33.6)	81 (33.8)	18 (32.7)	

On work engagement, 29 (16.6%) respondents scored low to extremely low (i.e., the 25^th^ groups’ percentile) and 97 (32.9%) respondents scored high to extremely high (i.e., the groups’ 75^th^ percentile).

When compared to the UBOS and UWES reference scores, the EMPs in this study did not score significantly different on emotional exhaustion scores (1.86 vs. 1.78, p = 0.31). The EMPs did score significantly lower on personal accomplishment (4.12 vs. 4.22, p = 0.03), but higher on overall work engagement (3.48 vs. 3.10, p < 0.01) (Table 2) [15–19].

**Table 2:** Burnout and work engagement scales compared to norm reference values of Dutch healthcare personnel. Burnout scales in reference to the validated norm reference values of Dutch healthcare personnel presented in the UBOS manual and Dutch physicians of the UWES manual [15–19]. * The UBOS manual applies gender-specific reference values to depersonalization scale due to consistently significant differences between male and female subjects, explained in terms of prevailing gender roles.

	Emergency physicians (n = 295)		Reference group (n = 10,552)			
Burnout scales	Mean	SD	Mean	SD	p	95% CI
Emotional exhaustion	1.86	1.15	1.78	1.21	0.31	−0.07 - 0.23
Depersonalization						
Women	1.21	0.78	1.14	0.78	0.12	−0.02 - 0.16
Men	1.45	1.11	1.27	0.84	<0.01	0.08 - 0.28
Diminished personal accomplishment	4.12	0.70	4.22	0.78	0.03	−0.19 - −0.01
	**Emergency physicians (n = 295)**		**Reference group (n = 655)**			
**Work engagement scales**	**Mean**	**SD**	**Mean**	**SD**	**p**	**95% CI**
Overall work engagement	3.48	0.65	3.10	0.87	<0.01	−0.61 - −0.16
Subscales						
Vigor	3.39	0.79	3.04	0.92	<0.01	−0.47 - −0.23
Dedication	3.78	0.81	3.29	1.04	<0.01	−0.62 - −0.36
Absorption	3.26	0.89	2.96	1.92	0.011	−0.53 - −0.07

### Intention to leave

Based on a Cronbach’s alpha of 0.62 – considered a weak internal consistency – the three questions were interpreted as assessing distinctly different aspects of the concept ofthe intention to leave and were assessed separately instead of taken together. Almost a third of respondents (29%) answered having considered leaving the profession several or multiple times. The consideration to leave the profession was significantly more common amongst respondents with burnout (n = 55, 36%) than those without (n = 240, 10%, p < 0.001). Sixty-eight respondents (23%) did not see themselves still working as EMP in 10 years’ time. This was also more common in physicians with burnout (49%) than those without (17%, p < 0.001). Physician age did not explain this difference (39.7 ± 5.7 vs 41.0 ± 5.8 years, 95% CI of difference −0.3 to 2.9 years, p = 0.103). Similarly, 23% of respondents reported not choosing EM as a career again (49% in burned out vs 18% in not burned out physicians, p < 0.001).

Table 3 shows the odds ratios from logistic regression analysis of burnout and work engagement on the intention to leave questions.

**Table 3:** Odds ratios (OR) of burnout and work engagement on the intention to leave questions.

	Positive future			Choosing EM again			Not considering to quit		
	OR	95% CI	p	OR	95% CI	p	OR	95% CI	p
Burnout	0.2	0.1 - 0.4	<.001	0.2	0.1 - 0.4	<.001	0.2	0.1 - 0.3	<.001
Work engagement	3.3	2.1 - 5.2	<.001	4.2	2.6 - 6.6	<.001	3.5	2.3 - 5.4	<.001

### Working conditions

Table 4 shows the adherence of respondents’ EDs to the 12 dichotomized workforce recommendations and their association with burnout. There were differences in the adherence to the recommendations. The mean of followed recommendations was 6.6 with a range of 1–11. The most often followed recommendation (99.0%) was to work with designated ED residents, while the least commonly followed recommendation (8.3%) was the use of a fixed cycle in rotation scheduling.

**Table 4:** Adherence in the respondents’ ED to the 12 workforce recommendations and association with burnout. Numbers are n (%). Chi-square tests.

Recommendation	Answers	N (%)	Burnout	No burnout	p
Scheduled backwards rotation	0-1x/month	220 (76.9%)	40 (18.2%)	180 (81.8%)	0.78
	>1/month	66 (23.1%)	13 (19.7%)	53 (80.3%)	
Fixed cycle in rotation scheduling	Yes	24 (8.3%)	7 (29.2%)	17 (70.8%)	0.17
	No	265 (91.7%)	47 (17.7%)	218 (82.3%)	
Fixed shift off per week	Yes	165 (56.3%)	26 (15.8%)	139 (84.2%)	0.17
	No	128 (43.7%)	29 (22.7%)	99 (77.3%)	
No break during shift	Never/sometimes	298 (58.8%)	8 (9.5%)	76 (90.5%)	0.01
	Often/always	209 (41.2%)	47 (20.9%)	162 (77.5%)	
Working full weekends per month	1 in 3–5 weekends	68 (23.2%)	8 (11.8%)	60 (88.2%)	0.09
	1 in 1–2 weekends	225 (76.8%)	47 (20.9%)	178 (79.1%)	
Appropriate team size for nightshifts	Yes	182 (66.2%)	29 (15.9%)	153 (84.1%)	0.25
	No (too small)	93 (33.8%)	20 (21.5%)	73 (78.5%)	
Shift duration 9 hours	Yes	180 (61.0%)	26 (14.4%)	154 (85.6%)	0.02
	No	115 (39.0%)	29 (25.2%)	86 (74.8%)	
Contract size per week	32-38 hours	208 (70.3%)	36 (17.3%)	172 (82.7%)	0.36
	<32 or>38 hours	88 (29.7%)	19 (21.8%)	68 (78.2%)	
Additional scheduled time	≥15%	63 (23.2%)	8 (12.7%)	55 (87.3%)	0.18
	<15%	209 (76.8%)	42 (20.1%)	167 (79.9%)	
Working with designated ED residents	Yes	292 (99.0%)	54 (18.5%)	238 (81.5%)	0.51
	No	3 (1.0%)	1 (33.3%)	2 (66.7%)	
Team size in relation to ED size	Sufficient	32 (11.9%)	5 (15.6%)	27 (84.4%)	0.82
	Insufficient	238 (88.1%)	41 (17.2%)	197 (82.8%)	
Contract type	Permanent	258 (87.5%)	43 (16.7%)	215 (83.3%)	0.02
	Temporary	37 (12.5%)	12 (32.4%)	25 (67.7%)	

### Associations

The higher the number of recommendations a respondent’s ED adhered to correlated weak, yet significantly with burnout (Spearman ρ = −.2, p < 0.01), with a positive future (ρ = .2, p < 0.01), with choosing EM again (ρ = .1, p = .04) and considering to quit (ρ = .2, p = 0.02). Three workforce recommendations showed weak but significant correlation on their own with an increased risk of burnout when not adhered to: the presence of breaks during shifts (ρ = −.2, p = .01), a shift duration of 9 hours (ρ = −.14, p = .02), and a permanent contract (ρ = −.13, p = .02) (Table 4).

Work engagement was associated with working 1 in 3–4 weekends or less (ρ = .2, p < 0.03). Intention to leave the profession was associated with not adhering to the following recommendations: breaktime (ρ = .2, p < .001), working 1 in 3–4 weekends or less (ρ = .2, p < 0.01), shift length (ρ = .1, p = .04) and working in a team large enough to match the ED size (ρ = .1, p = 0.05).

## Discussion

### Main findings and interpretation

In this study, we examined adherence to Dutch workforce guidelines for EMPs and investigated the association between compliance with these recommendations and levels of burnout, work engagement and intention to leave the profession. Adherence to workforce recommendations varied with a mean of only 6.6 followed: the recommendation to work with designated ED residents was followed most frequently (99.0%), while the use of a fixed cycle in rotation scheduling was adhered to least (8.3%). Not adhering to three specific workforce recommendations was significantly associated with burnout: the absence of breaks during shifts, a shift duration of less or more than 9 hours, and a temporary contract. Work engagement was associated with working 1 in 3–4 weekends or less. Intention to leave the profession was associated with not adhering to the following recommendations: having breaktime, working 1 in 3–4 weekends or less, shift length of 9 hours and working in a team large enough to match the ED size. Although these relationships are weak and cannot be interpreted as causal, they do provide a basis for further investigation of the hypothesis that with better implementation, adherence to a workforce guideline aimed at durable employment may protect against burnout, job turnover and may enhance work engagement.

Comparing the prevalence of burnout symptoms in our population of Dutch EMPs (18.6%) with that reported in other studies using the Maslach burnout inventory proved less straightforward than expected, mainly due to the use of different cut-off values [22]. The study of Meynaar et al from 2015 showed a comparable way of calculating burnout, but reported a surprising low prevalence of 4.4% among 272 Dutch intensivists (41% response rate), which the authors hypothesised was due to the low workload in Dutch ICUs [23]. A previous meta-analysis, including over 1250 EMPs from 12 countries, showed high levels of emotional exhaustion and depersonalization (both around 40%) and low personal accomplishment in 35% of EMPs [24]. While these numbers are higher than those observed in our study, they are not directly comparable due to the use of various different cut-offs. Despite our relatively favourable results, we must recognize that 1 in 5 Dutch EMPs in our study displayed signs of burnout. Given the potential negative consequences for both physicians and patients, this prevalence is concerning and underscores the need for intervention [9–11]. Adhering to the workforce recommendations presents one avenue for improvement.

Work engagement (3.48, SD 0.65) was reported higher in Dutch EMPs compared to other Dutch medical specialists (3.10, SD 0.87) as reported by the UWES manual [19]. Also compared to the UWES-3 scores on work engagement of 5.023 direct care nurses from 6 European countries the Dutch EMPs scored higher [25]. This might be explained by emergency medicine in The Netherlands being a still relatively young medical specialty with lots to develop and proof, and a dynamic, exhilarating workload.

Since our study is the first to investigate the adherence to a workforce guideline, direct comparisons with other studies is challenging. Nevertheless, the workforce recommendations are based on previously published research and similar strategies for preventing burnout have been reported by others [2–4,13,26–28]. Time during a shift to take a break, recover and have designated time to eat are all proven to be beneficial for physician wellness [28–32]. Working 9 hours provides overlap between colleagues to safely transfer care of patients and to end the shift before concentration and energy levels diminish [32]. The job security that comes with a permanent contract besides the impact it has on team building might explain this recommendation to be associated with burnout. Having a sufficient team size helps to distribute workload.

### Strengths and limitations

Our study is the first to investigate the prevalence of burnout, work engagement and intention to leave among Dutch EMPs. Also, a workforce guideline such as the DSEP guideline, even though based upon numerous studies, has not been analysed for its effects on these three outcomes as a set of recommendations. The response rate was 56% which we consider high in comparison with similar survey studies among physicians. The use of validated instruments, such as the Dutch version of the Maslach Burnout Inventory (UBOS) and the Utrecht Work Engagement Scales (UWES) allows for future comparisons.

The adherence to the workforce recommendations proved mediocre, variation was high, and only some recommendations showed (weak but significant) correlation. No adjustment for confounders at this point was made. Nevertheless, this study creates a foundation for future research and efforts to promote a sustainable EM workforce. This study did not account for the potential effects of the COVID-19 pandemic. Therefore, repeating this research in the current Dutch EM environment is recommended.

## Conclusion

We found that almost 1 in 5 Dutch EMPs exhibited signs of burnout, just prior to the COVID-19 pandemic. On the opposite, levels of work engagement were higher compared to other Dutch medical specialists. Adherence to the DSEP workforce recommendations promises to be an effective strategy to prevent burnout and job turnover, and promote work engagement. Five recommendations in particular deserve to be addressed: the presence of recovery time during shifts, a shift duration of 9 hours, working less weekends, sufficient team size and the availability of a permanent contract. Repeating this study to obtain updated prevalence rates is planned, concurrent with the publication of the revised Workforce guideline. The results of this current study were used in its revision and provide the basis of a longitudinal analysis. It is crucial for a sustainable career in EM to create better work conditions and terms of employment for health care professionals.

## Supporting information

S1 Table Questionnaire workforce article English PLOS one. (DOCX)

S2 Table Dutch Society of Emergency Physicians table of members 2019. (XLSX)
