# Supplementary material for: Workforce guidelines, burnout, work engagement and the intention to leave: A cross-sectional study among Dutch emergency physicians
Source: PLoS One. 2026 Oct 5;21(10):e0337882. doi: 10.1371/journal.pone.0337882 (PMC13637872; doi:10.1371/journal.pone.0337882)
Supplement: Supplementary file 1 [file pone.0337882.s001.docx]

SUPPLEMENTARY DIGITAL CONTENT

Supplementary Online Content – Questionnaire_Dutch.docx

| **NR** | **ITEMS** | **QUESTION** | **ANSWERS** |
| --- | --- | --- | --- |
| ***BACKGROUND*** | | |  |
| 1 | **1** | What is your year of birth? | Open {year} |
| 2 | **1** | What is your gender? | {1,male}  {2,female} |
| 3 | **1** | What is your marital status? | {1,Living together or married, with children}  {2,Living together or married, without children}  {3,Single, with children}  {4,Single, without children}  {5,Other, namely:} |
| 3b |  | What is your marital status? – Other, namely | Open {text} |
| 4 | **4** | Can you indicate how many people are in your household? | Open {number} |
|  | 4-A | Number of adults | |
|  | 4-B | Number of children 0-3 years | |
|  | 4-C | Number of children 4-12 years | |
|  | 4-D | Number of children 13-18 years | |
| 5 | **1** | In which year did you graduate medical school? | Open {year} |
| 6 | **1** | In which year did you graduate as an emergency physician? | Open {year} |
| CONTRACT | | |  |
| 7 | **1** | In what type of hospital are you currently working? | {1,University Medical Center}  {2,Collaborating Top Clinical Hospitals}  {3,Collaborating General Hospitals}  {4,Abroad}  {5,Not working as an emergency physician, including e.g. sabbatical, full sick leave} |
| 7a | **1** | Following answer 7 - {5}  Can you indicate why you are currently not working as an emergency physician? | {1,Sick leave, not working}  {2,I have taken a temporary break (for example, sabbatical)}  {3,I have left my profession as an emergency physician}  {4,I am doing research}  {5,I have a (temporary) different position, but will return to work as an emergency physician in the future}  {6,Other, namely} |
| 7b |  | Following answer 7a - {6}  If you are currently not working as an emergency physician, can you indicate why - Other, namely | Open {text} |
| 9 | **1** | My contract falls under: | {1,CAO - Hospitals}  {2,CAO - AMS}  {3,CAO - University Medical Center}  {4,Only self-employed / temporary worker}  {5,A combination of self-employed / temporary worker, and CBA}  {6,Other} |
| 9b |  | Following 9 – {6} Other, namely | Open {text} |
| 9c |  | Following 9 – {4-5} How many hours do you work on average per month as a self-employed / temporary worker? | Open {hours} |
| 10 | **1** | Do you have a temporary contract or a permanent contract? | {1,Temporary}  {2,Permanent} |
| 11 | **2** | How many hours do you work contractually (on paper) per week? | Open {hours} |
| 11b |  | Are you currently working on a therapeutic basis? | {0,No}  {1,Yes} |
| 12 | **1** | What is the formative size of your contract expressed in fte (full-time equivalent)? | Open {number 0-1}  {999,Not applicable} |
| 13 | **1** | How many hours do you actually work, including overtime, on average per week? | Open {hours} |
| YOUR WORK AND RESPONSIBILITIES | | |  |
| 14 | **1** | What is the current formation of emergency physicians in number of persons? | Open {number}  {999,Not applicable} |
| 15 | **1** | What is the current formation of emergency physicians in fte? | Open {number}  {999,Not applicable} |
| 16 | **1** | Does the emergency department where you work receive more than 18,000 patients per year? | {0,No}  {1,Yes}  {999,Not applicable} |
| 17 | **1** | Are you currently active as the principal investigator of a scientific study? | {1,No, and I have never conducted scientific research either}  {2,No, I have conducted scientific research, but I am not currently actively conducting scientific research}  {3,No, I have obtained my PhD, but I am not currently conducting active scientific research}  {4,Yes, I am actively conducting scientific research, but not related to a promotion track}  {5,Yes, I am working on a promotion track}  {6,Yes, I am actively conducting scientific research and I have been promoted} |
| 18 | **1** | How many hours do you spend on research on average per month? | Open {hours} |
| 19 | **3** | To what degree of independence are the following procedures performed by the emergency physician? It is assumed that in case of doubt regarding the procedure, you always consult with a colleague specialist. |  |
|  | 19-A | Procedural Analgesia and Sedation (PSA) | {1,Independently}  {2,Under bedside supervision of a colleague specialist}  {3,Not myself, done by a sedation team in the emergency department}  {4,Not myself, takes place in the operating room (OR)} |
|  | 19-B | Emergency Ultrasound (eFAST, RUSH, etc) | {1,Independently}  {2,Independently, but when indicated for (emergency) ultrasound, the images are always repeated by a radiologist (in training)}  {3,Under bedside supervision of a colleague specialist}  {4,No, done by the radiologist (in training)} |
|  | 19-C | (Emergency) intubation | {1,Independently}  {2,Under bedside supervision of a colleague specialist}  {3,Not myself, usually done by the anesthesiologist or intensivist in the emergency department}  {4,Not myself, takes place in the operating room (OR) or Intensive Care (IC)} |
| 20 | **9** | Can you check who falls under your supervision in the emergency department? Multiple answers possible. | {0,No}  {1,Yes} |
|  | 20-A | Nobody | |
|  | 20-B | Emergency Medicine Resident | |
|  | 20-C | Residents from other disciplines | |
|  | 20-D | Junior Doctor | |
|  | 20-E | Trainee Doctor | |
|  | 20-F | Assistant Medical Officer | |
|  | 20-G | Physician Assistants, Nurse Specialists | |
|  | 20-H | Co-assistant | |
|  | 20-I | Senior co-assistant / semi-doctor |  |
| 21 | **1** | As an emergency physician, I have final responsibility / primary care responsibility for: | {1,All emergency patients}  {2,All self-referrals}  {3,All self-referrals and 112 cases}  {4,All self-referrals, the 112 cases, and an agreed group of referred patients}  {5,None of the emergency patients}  {6,Other} |
| 21b |  | Following 21 – {6} As an emergency physician, I have final responsibility / primary care responsibility for: - other, namely | Open {text} |
| 23 | **5** | With the following question, we want to challenge you to outline your ideal emergency care situation, assuming that anything is possible. | {1,Completely disagree}  {2,Disagree}  {3,Neither agree nor disagree}  {4,Agree}  {5,Completely agree} |
|  | 23-A | Every emergency patient must be treated by an emergency physician. | |
|  | 23-B | The emergency physician only provides supervision and bedside guidance. | |
|  | 23-C | The emergency physician is primary responsible for every emergency patient. | |
| SCHEDULE | | |  |
| 24 | **1** | How much scheduled time do you get for non-clinical work, also known as additional differentiated work (AGI)? | Open {hours}  {999,Not applicable} |
| 25 | **1** | Scheduled backward rotation, where there is less than 24 hours between the start times of shifts, occurs with us: (Note separate from self-swapped shifts) | {1,Never}  {2,Less than 1 shift every few months}  {3,About 1 shift per month}  {4,About 2-4 shifts per month}  {5,Weekly}  {999,Not applicable} |
| 26 | **1** | Our schedule is based on a fixed cycle of shifts, resulting in a predictable schedule pattern. | {0,No}  {1,Yes}  {999,Not applicable} |
| 27 | **1** | I have a fixed day/evening off each week. | {0,No}  {1,Yes} |
| 28 | **1** | I work on average X weekends per month. | Open {0-5} |
| 29 | **1** | How many evening shifts do you work on average per month? | Open {number} |
| 30 | **1** | How many night shifts do you work on average per month? | Open {number} |
| 31 | **1** | How many hours is the longest scheduled shift for you, including breaks? | Open {hours} |
| STRESSORS | | |  |
| 32 | **4** | Work pressure | {1,Never}  {2,Sometimes}  {3,Regularly}  {4,Often}  {5,Always} |
|  | 32-A | Do you have too much work to do? | |
|  | 32-B | Do you have to work very quickly? | |
|  | 32-C | Are you interrupted during your work? | |
|  | 32-D | Do you have to do too many things at once? | |
| 33 | **6** | Work recovery time | {1,Never}  {2,Sometimes}  {3,Regularly}  {4,Often}  {5,Always} |
|  | 33-A | During my shift, I have to skip my break | |
|  | 33-B | I can leave the department during my break | |
|  | 33-C | During my shift, there is room for a social conversation with colleagues or others | |
|  | 33-D | After a heavy case, I feel sufficiently recovered when I go to the next patient | |
|  | 33-E | After a heavy case, there is an official debriefing | |
|  | 33-F | After a heavy case, there is room for informal debriefing | |
| 34 | **4** | Work-life conflict | {1,Never}  {2,Sometimes}  {3,Regularly}  {4,Often}  {5,Always} |
|  | 34-A | Do you experience problems combining work and private life? | |
|  | 34-B | How often do you have to cancel or refuse a private appointment because of your work? | |
|  | 34-C | How often do you have to cancel a private appointment because you are exhausted? | |
|  | 34-D | How often do you do work tasks at home? | |
| 35 | **1** | Is your work emotionally demanding? | {1,Never}  {2,Sometimes}  {3,Regularly}  {4,Often}  {5,Always} |
| 36 | **2** | Misuse | {1,Never}  {2,Sometimes}  {3,Regularly}  {4,Often}  {5,Always} |
|  | 36-A | I spend time and energy on patients in the ER without an acute care need, so patients who do not belong in the ER | |
|  | 36-B | My administrative burden is too high | |
| 37 | **2** | Work tasks | {1,Never}  {2,Sometimes}  {3,Regularly}  {4,Often}  {5,Always} |
|  | 37-A | I find it difficult to combine my tasks and responsibilities as an ER doctor with those as a trainer | |
|  | 37-B | How often do you feel that you need to intervene in the diagnostic and therapeutic actions of a medical assistant? | |
| 38 | **6** | In the past 12 months, have you personally encountered unwanted behaviors at work, as listed below? | {1,Never}  {2,Sometimes}  {3,Regularly}  {4,Often}  {5,Always}  Or  {0,No}  {1,Yes} |
|  | 38-A | Bullying | |
|  | 38-B | Discrimination | |
|  | 38-C | Threat / aggression | |
|  | 38-D | (sexual) Harassment | |
|  | 38-E | Physical or psychological boundary-crossing behavior | |
|  | 38-F | Social exclusion / being excluded | |
| ENERGY SOURCES | | |  |
| 39 | **3** | Learning and development opportunities | {1,Completely disagree}  {2,Disagree}  {3,Neither agree nor disagree}  {4,Agree}  {5,Completely agree} |
|  | 39-A | I keep learning new things at my job | |
|  | 39-B | In my current job, I can develop my strengths | |
|  | 39-C | My job offers sufficient opportunities for personal growth and development | |
| 40 | **2** | Does the work suit you? | {1,Completely disagree}  {2,Disagree}  {3,Neither agree nor disagree}  {4,Agree}  {5,Completely agree} |
|  | 40-A | My current job fits well with what I can do / my skills | |
|  | 40-B | My current job fits well with what I want / my abilities | |
| 41 | **5** | Room for maneuver and autonomy | {1,Never}  {2,Sometimes}  {3,Regularly}  {4,Often}  {5,Always} |
|  | 41-A | My work gives me the space to make my own decisions | |
|  | 41-B | I determine the way I perform my work myself | |
|  | 41-C | I participate in decisions about organizational and logistical issues | |
|  | 41-D | I determine when I perform my tasks myself | |
|  | 41-E | I can determine the content of my work myself | |
| 42 | **4** | Support from your colleagues in the ER;  This refers to the caregivers you work with during a shift and for example, take care of an acutely ill patient | {1,Never}  {2,Sometimes}  {3,Regularly}  {4,Often}  {5,Always} |
|  | 42-A | Can you ask your colleagues in the ER for help when needed? | |
|  | 42-B | Do you feel recognized and valued in your work by your colleagues in the ER? | |
|  | 42-C | Are your colleagues in the ER accessible for discussing both personal and business matters? | |
|  | 42-D | Do you feel recognized and valued in your work by your fellow medical specialists? | |
| 43 | **3** | Team atmosphere.  By "team" we mean the group of caregivers you work with during a shift and for example, take care of an acutely ill patient | {1,Never}  {2,Sometimes}  {3,Regularly}  {4,Often}  {5,Always} |
|  | 43-A | Do you feel comfortable in your team? | |
|  | 43-B | Is there a pleasant atmosphere in your team? | |
|  | 43-C | Can you openly and honestly discuss mistakes made in your team? | |
| 44 | **2** | Relationship with patients | {1,Very little}  {2,Little}  {3,Not much / not little}  {4,Much}  {5,Very much} |
|  | 44-A | How much do you invest in your patients (e.g. attention, time, energy, effort)? | |
|  | 44-B | How much do you receive back from them (e.g. appreciation, recognition, gratitude)? | |
| 45 | **4** | Relationship with your organization | {1,Very little}  {2,Little}  {3,Not much / not little}  {4,Much}  {5,Very much} |
|  | 45-A | How much do you invest in your organization (e.g. attention, time, energy, effort)? | |
|  | 45-B | How much do you receive back from your organization (e.g. appreciation, recognition, gratitude)? | |
|  | 45-C | The organization pays attention to the mental burden that my work has on me | |
|  | 45-D | The organization pays attention to the physical burden that my work has on me | |
| 46 | **3** | The functioning of your team.  By "team" we mean the group of caregivers you work with during a shift and for example, take care of an acutely ill patient | {1,Never}  {2,Sometimes}  {3,Regularly}  {4,Often}  {5,Always} |
|  | 46-A | Do you work effectively together in your team? | |
|  | 46-B | Are the work agreements in your team being followed well? | |
|  | 46-C | Does everyone do their best in your team? | |
| WELL-BEING – Burnout - UBOS-C | | |  |
| 47 | **8** | Your stress at work  W_UBOSU | {1,Never}  {2,Sporadically}  {3,Occasionally}  {4,Regularly}  {5,Often}  {6,Very often}  {7,Always} |
|  | 47-A | I feel mentally exhausted by my work. | |
|  | 47-B | Working a whole shift is a heavy burden for me. | |
|  | 47-C | I feel "burned out" by my work. | |
|  | 47-D | At the end of a workday, I feel empty. | |
|  | 47-E | I feel tired when I get up in the morning and there is another workday ahead of me. | |
|  | 47-F | I feel frustrated by my job. | |
|  | 47-G | I think I invest too much in my work. | |
|  | 47-H | I feel at the end of my rope. | |
| 48 | **5** | Your stress at work  W_UBOSD | {1,Never}  {2,Sporadically}  {3,Occasionally}  {4,Regularly}  {5,Often}  {6,Very often}  {7,Always} |
|  | 48-A | I feel that I treat some patients too impersonally. | |
|  | 48-B | I feel that I have become more indifferent towards other people since I got this job. | |
|  | 48-C | I worry that my work dulls my emotions. | |
|  | 48-D | I really don't care what happens to some patients. | |
|  | 48-E | I feel that patients blame me for their problems. | |
| 49 | **6** | Your stress at work  W_UBOSC | {1,Never}  {2,Sporadically}  {3,Occasionally}  {4,Regularly}  {5,Often}  {6,Very often}  {7,Always} |
|  | 49-A | I can easily feel empathy for my patients. | |
|  | 49-B | I know how to adequately solve the problems of the patients. | |
|  | 49-C | I feel that I positively influence the lives of other people through my work. | |
|  | 49-D | I can easily create a relaxed atmosphere with my patients. | |
|  | 49-E | Working with patients cheers me up. | |
|  | 49-F | I have achieved many valuable things in this job. | |
|  | 49-G | In my work, I handle emotional problems very calmly. | |
| 50 | **3** | Work engagement | {1,Never}  {2,Sometimes}  {3,Regularly}  {4,Often}  {5,Always} |
|  | 50-A | When I work, I feel fit and strong | |
|  | 50-B | I am enthusiastic about my work | |
|  | 50-C | I completely immerse myself in my work | |
| 51 | **3** | Sleep quality  “In the last 3 months, I have...” | {1,Never}  {2,Sometimes}  {3,Regularly}  {4,Often}  {5,Always} |
|  | 51-A | Difficulty falling asleep | |
|  | 51-B | Difficulty staying asleep | |
|  | 51-C | Trouble with sleepwalking, nightmares,  panic attacks, teeth grinding, and/or restlessness in the legs | |
| 52 | **3** | General well-being  “In the past month, I have...” | {1,Never}  {2,Sometimes}  {3,Regularly}  {4,Often}  {5,Always} |
|  | 52-A | …suffered from tension complaints such as headaches, dizziness, muscle pain, palpitations, and nausea | |
|  | 52-B | …felt unhappy and depressed | |
|  | 52-C | …been able to enjoy my ordinary, daily activities | |
| 53 | **1** | What do you think, overall, about your health? | {1,Poor}  {2,Moderate}  {3,Sufficient}  {4,Good}  {5,Very good} |
| 54 | **1** | Have you ever had a burnout? | {1,No}  {2,Yes. At that time, I was not yet working as an emergency physician}  {3,Yes. At that time, I was already working as an emergency physician} |
| 55 | **1** | Can you indicate how often you reported being sick or absent in the past year? | {0,0 times}  {1,1-3 times}  {2,4-7 times}  {3,8 or more times} |
| 56 | **1** | Can you indicate how many days you stayed home in the past year due to illness or problems? | {0,0 days}  {1,1 to 6 days}  {2,7 to 14 days}  {3,15 to 30 days}  {4,1 to 3 months}  {5,Longer than 3 months} |
| 57 | **1** | How often has it occurred in the past year that you went to work while you were actually sick? | {0,0 times}  {1,1-3 times}  {2,4-7 times}  {3,8 or more times} |
| 58 | **1** | All things considered: how happy are you with your life?  (school grade 1-10) | Open {number} |
| PERSONAL RESOURCES | | |  |
| 59 | **3** | Flexibility | {1,Completely disagree}  {2,Disagree}  {3,Neither agree nor disagree}  {4,Agree}  {5,Completely agree} |
|  | 59-A | If the work requires it, I am willing to change my schedule. | |
|  | 59-B | I easily adapt to changes at work. | |
|  | 59-C | I have no trouble changing my working method. | |
| 60 | **3** | Setting boundaries | {1,Completely disagree}  {2,Disagree}  {3,Neither agree nor disagree}  {4,Agree}  {5,Completely agree} |
|  | 60-A | I carefully guard my own boundaries. | |
|  | 60-B | As soon as I notice that I am crossing my own boundaries, I take a step back. | |
|  | 60-C | Sometimes I also say "no" at work. | |
| 61 | **5** | Belief in one's own abilities | {1,Completely disagree}  {2,Disagree}  {3,Neither agree nor disagree}  {4,Agree}  {5,Completely agree} |
|  | 61-A | If I am called upon in different ways at the same time, I can handle it well. | |
|  | 61-B | I am good at making difficult decisions or many decisions at the same time. | |
|  | 61-C | I manage to make emergency care run efficiently. | |
|  | 61-D | I manage to make emergency care run carefully. | |
|  | 61-E | I am an expert in emergency care. | |
| 62 | **4** | Recovery in free time | {1,Never}  {2,Sometimes}  {3,Regularly}  {4,Often}  {5,Always} |
|  | 62-A | In my free time, I forget my work. | |
|  | 62-B | In my free time, I do relaxing activities. | |
|  | 62-C | In my free time, I have energy and time for things that broaden my horizon | |
|  | 62-D | In my free time, I determine for myself how I want to spend my time. | |
| 63 | **4** | Perfectionism | {1,Completely disagree}  {2,Disagree}  {3,Neither agree nor disagree}  {4,Agree}  {5,Completely agree} |
|  | 63-A | I am a perfectionist. |  |
|  | 63-B | I often correct the last mistakes in my colleagues' work. |  |
|  | 63-C | Tasks I assign must be carried out exactly as I have said. |  |
|  | 63-D | I strive to do my work perfectly. |  |
| ORGANIZATIONAL OUTCOMES | | |  |
| 64 | **1** | Have you ever seriously considered quitting your job as an emergency physician? | {0,Never happened}  {1,Once happened}  {2,Several times happened}  {3,Multiple times happened} |
| 65 | **8** | What reasons played a role at the time you considered quitting? (multiple answers possible) | {0,No}  {1,Yes} |
|  | 65-A | my work did not meet expectations in terms of content. | |
|  | 65-B | my work did not meet expectations in terms of hospital or departmental culture. | |
|  | 65-C | there was too much conflict and/or insufficient support for emergency physicians. | |
|  | 66-D | I experienced the workload as too high. | |
|  | 65-E | due to a disciplinary case, incident, or complaint. | |
|  | 65-F | my work-life balance was disrupted. | |
|  | 65-G | stagnant development | |
|  | 65-H | another reason, namely | |
| 65b |  | What reasons played a role at the moment you considered quitting?  – Other, namely | Open{text} |
| 66 | **1** | Would you choose this profession again with the knowledge you have now? | {0,No}  {1,Yes} |
| 67 | **1** | Do you see yourself still working as an emergency physician in 10 years? | {0,No}  {1,Yes} |

You have reached the end of the survey. On behalf of the NVSHA, the section of Arts and Welfare, and the research group, thank you very much for participating in the research.

Your individual report is already ready. You can now view your own scores. Click on "next" to go to the first page of this report. From that page, you can also download a .pdf of your report. If you prefer to look at your report at a later time, you can do so via the link that will be sent to you via your email address when you complete this survey.

Note: If you proceed to your personal report now, you will not be able to change your answers to the survey questions. If you have questions about the execution of this scan, you can send a message to [info@3ihc.nl](mailto:info@3ihc.nl). If you have questions about the underlying research, you can send a message to burnout@nvsha.nl.
