# Supplementary material for: Workforce guidelines, burnout, work engagement and the intention to leave: A cross-sectional study among Dutch emergency physicians
Source: PLoS One. 2026 Oct 5;21(10):e0337882. doi: 10.1371/journal.pone.0337882 (PMC13637872; doi:10.1371/journal.pone.0337882)
Supplement: Supplementary file 2 [file pone.0337882.s004.docx]

PONE-D-25-60264

Workforce guidelines, burnout, work engagement and the intention to leave. A cohort study among Dutch Emergency Physicians.

PLOS ONE

**Response to reviewers**

Dear Editor,

We appreciate the reviewers' valuable comments on our paper ‘Workforce guidelines, burnout, work engagement and the intention to leave. A cohort study among Dutch Emergency Physicians’. We have thoroughly studied the comments and are pleased to provide the following answers and corresponding changes in our paper. We will be pleased to further elaborate, or adjust the manuscript if any of the comments were not sufficiently implemented or explained.

| **Reviewer #1 Comments** | **Revisions (location of revisions in clean manuscript)** | **Page number, line number** |
| --- | --- | --- |
| The manuscript is well conducted, clearly written, and methodologically sound. The use of validated instruments (UBOS and UWES) is appropriate, and the statistical analyses are rigorous. The data support the authors’ conclusions and provide a meaningful contribution to understanding the well-being of emergency physicians in the Netherlands. | We thank the reviewer for the compliments. We appreciate the acknowledgement of the quality of our work. | NA |
| However, it is important to emphasize that the statistical correlations identified do not imply causality. They indicate significant associations between workforce recommendations and levels of burnout, work engagement, and intention to leave, but these relationships should not be interpreted as causal. Clarifying this point in the text would help prevent deterministic interpretations. | Thank you. We fully agree with the reviewer’s comment. We reviewed the texts to see where we may have caused any confusion. We chose to make a specific statement in the Discussion – Main findings section. | Page 10, line 285-288 |
| Additionally, while the questionnaires used are validated and reliable, the burnout measure does not constitute a formal clinical diagnosis. The results should be interpreted as an indicator of burnout tendency or risk, rather than confirmed burnout, which would require assessment by qualified medical or psychological professionals. A short clarification in the methods or discussion would strengthen the accuracy of interpretation. | Valid point. The burnout tool does not measure burnout as a diagnosis on a personal level. We went through the text to ascertain if at any point the text may suggest burnout to be a diagnosis, and we added a clarification in the method section of our paper. | Page 3  Line 67  Page 4 line 115  Page 5  Line 146, 150-151, 154-155  Page 10, line 289 |
| Finally, as this study was conducted before the COVID-19 pandemic, its findings provide a valuable baseline but may not reflect current realities. Given the substantial changes in emergency care systems worldwide, a follow-up or pre-post study design would be highly relevant to assess potential shifts in burnout and work engagement after the pandemic. | We have found this a valid point as well and are pleased to announce we are currently collecting data in the follow-up study.  In its first set-up, a follow-up study or longitudinal approach was envisioned, but aimed at the workforce guideline and its adherence. The guideline tested stems from 2015 and a revision was planned for 2020. Hence, the study from 2019 was set to be a baseline and a provider of data for the rewrite. The guideline revision was delayed due to a number of causes, including the COVID-19 pandemic and the recognition of Emergency Medicine as a medical specialty in the Netherlands. The revision has been published in December 2025 and the follow-up study is currently ongoing. | NA |
| Overall, this is a well-designed and valuable study that addresses an important topic. The manuscript would benefit from the clarifications above, which would enhance its methodological precision and interpretative transparency. | We thank the reviewer for the opportunity to share our study and address the above mentioned clarifications. We hope to have addressed them to satisfaction. | NA |
| **Reviewer # 2 Comments** |  |  |
| Generally, your manuscript is easily legible, well structured and straightforward in its aims, results and discussion. | We thank the reviewer for the compliments. We appreciate the acknowledgement of the quality of our work. | NA |
| In my view, more elaborate statistical analyses would have been more interesting to the explanation of variance in your outcomes.  However, I guess you might have wanted to save these more extensive analyses for the longitudinal data. | Important remark and indeed, most of the focus on variance, and more sturdy predictors will be done in the follow-up study with the longitudinal data. | NA |
| Abstract - Please add the following information to the abstract: year of study, mode of survey administration*,* statistical procedures for data analysis, statistical program for data analysis. | Thank you for these practical tips on improving our abstract.  Abstract – methods line 48 now gives more information on mode of survey, and year. A new sentence is added about the statistical procedures. | Page 3, line 48. Line 51-53 |
| - Please write out abbreviations, e.g. DSEP*.* | The text now explains the DSEP abbreviation; a small omission on our part. | Page 3 line 45 |
| - In your Conclusions section, you repeat the findings from your Results section. I wonder if it would be more interesting to readers to see suggestions how a “decisive action” might look like instead, i.e. ideas on who could address the study results and which strategies might be plausible. | Abstract – conclusion. We removed the repeat of results, to make room for a more detailed recommendation. | Page 3 Line 68-72 |
| - I would suggest to reconsider pointing out the repeat survey in the abstract. By the time this paper is published, readers might wonder why authors did not prefer to wait for the study data from this successive study to analyze longitudinal data instead of cross-sectional study results. | The mention of the repeat study is removed from the abstract as suggested. | Page 3  Line 72 |
| Background - The Background section is rather short. Instead of or in addition the findings on the repercussions of burnout for healthcare providers in general, you might refer to empirical findings specifically for EM physicians to establish specific relevance for this professional group. This might also “complete the circle” with regard to the workforce guideline, i.e. why are certain recommendations included in this guideline. The same suggestion applies for work engagement. | The suggestion the reviewer gives is a valid one. However, there are no findings specific for EMPs on both burnout and work engagement, other than that EMPs overall almost ‘always’ score worst among peers. This is likely mainly due to the 24/7 schedule, in a fast-paced, unpredictable setting, where stressful situations are common. We rewrote the first sentence of the introduction to highlight these differences a bit more. | Page 4, line 87-88 |
| - Please add that you measured self-reported adherence and compliance to recommendations. Otherwise, one might confuse it with objective assessments of adherence to recommendations. | ‘Self -reported’ was added line 116, page 4 | Page 4 line 116 |
| Methods - Please clearly state which part of the study was cross-sectional and which is the cohort study (since a study cannot be cross-sectional and a cohort at the same time). I guess that you analyzed cross-sectional data from one wave of a cohort study. Please explain how many waves were already conducted and how many waves are planned. | This study was the first cross-sectional wave of a cohort study of which the second wave was planned for 5 years later. This has been explained more correctly now on page 5, line 123. | Page 1 Title  Page 4, line 115  Page 5, line 123 |
| **-** Please shortly explain how you gained access to the email addresses. I guess that you obtained them from the Dutch Society of Emergency Physicians? It would be helpful for international readers to know if every single Dutch emergency physician has to register with the Dutch Society of Emergency Physicians. If not, how many EM physicians are there in the Netherlands in total and how do they compare with those who are not part of the society? Are only emergency physicians working in hospitals eligible to participate or also those working in pre-hospital settings? | The Dutch RGS, the registration commission for medical specialists, had 583 registered EMPs at 01-01-2019. At 01-06-2019 DSEP confirmed us to have 526 members. 526/583 equals 90.2% of all Dutch EMPs. Most residencies end in September with approximately 45 new EMPs finishing their training. At 01-01-2020 there were 611 registered EMPs at the RGS, and 603 EMP members of DSEP. 98,7%. We added this in the text.  In the Netherlands all EMP work in EDs as a main part of their job. Prehospital care is not part of our job description.  Both facts are now clarified in the text. | Page 5 line 124-126 |
| - Did you apply any inclusion or exclusion criteria for participants? If yes, please explain. | The study included all Dutch EMPs registered with the DSEP. We added as per your suggestion a sentence to clarify this in the Analysis and statistics section of the methods. | Page 6, line 189-194 |
| - Please explain why you choose this specific set of demographic variables and not others from your comprehensive questionnaire, which might have also been suitable with regard to burnout (e.g. working hours (item 13), weekend work (item 28))? | We are unsure which section of text is meant by this comment. We are happy to clarify our choices, but would like to know where specifically the reviewer’s question is applicable to. | NA |
| - Please name the amount of items for each burnout domain since your participants answered these items and not the domains per se. | The amount of items per burnout domain were added to the text. | Page 5, line 144-145 |
| Why did you use two operationalizations of burnout, i.e. Brenninkmeijer EEplus1 definition and the one provided by the UBOS manual? | In literature on burnout the use of the MBI / UBOS is standard. Yet the cut-off for how and when to call cases positive or negative for burn-out symptomatology is varying highly.  Some use only the emotional exhaustion scale (EE), and leave out the other two domains (DP and PA) completely, other use a combination of two out of three domains, and none use the exact same cut-off. In general, studies on burnout report burnout rates going from 20 percent to as high as 90 percent, using the same measurement tool, but different interpretation of the outcomes.  Brenninkmeijers’ proposed EEplus1 (high EE and either high DP ór low PA) and the proposed Burnout Syndrome (both high EE ánd high DP plús low PA) are examples of unifying the way we report on burnout. To determine ‘high EE and DP, and low PA’ the manual suggests to use the 75^th^ percentile of the distribution of the composite variables.  We believe this way we report the most truthful representation of burnout symptoms in our sample. | Page 5  Line 142-155 |
| - What was the time horizon given in the question on “intention to quit”? Please add this information to the text. | We added this information to the text. In short: having ‘ever’ considered to leave the profession. | Page 6  Line 169-170 |
| - Please report the Cronbach’s alpha for all of your scales. Please also consider that CA is not a simple measure of “correlation” (page 7) but one of internal consistency. | We thank you for bringing it to our attention that we used the word correlation in the explanation of the Cronbach’s alpha, instead of internal consistency. The CAs were added. | Page 7, line 206-208 |
| - I could not find Figure 1 referenced on page 5. Please clarify. | The absence of figure 1 you so rightfully point out, is due to one of the final changes we had to make to our article to fit it within the set boundaries for submissions. So sorry, we forgot to remove the reference ‘figure one’. | Page 6 line 175 |
| - Please add information on how you addressed missing data in analyses. | We added transparency on how we dealt with missing data. The on line questionnaire worked in a way where respondents could only more forward after filling in all previous questions, so missing items was extremely rare. | Page 6, line 189-194 |
| - Please explain why you refrained from multivariable logistic regression analyses to include all demographic factors in one analytical model to explain variance in your outcomes? | A multivariate logistic regression analysis was performed correcting for age, sex, career length, and the most common work stressors and energy sources. These work stressors and energy sources were derived from the job demands and resources model (Schaufeli W, Bakker A, 2004). This model uses work pressure, role conflict and emotional burden as three well established job demands, and possibilities for personal growth, work fit, autonomy, peer support, team atmosphere and team effectiveness as six well known job resources.  For burnout as expected energy sources ( OR 0.8, with 0.7-0.9 CI 95% and p .003) and work stressors (OR 1.9 and 1.4-2.5 CI 95% at p <.0001) proved to be strong predictors. We tested the composite variable of the 12 workforce factors, which showed a calculated OR of 0.9 with 0.9-1.1 CI 95% for burnout.  Only the energy sources were of predictive value for work engagement (workforce came at OR 0.9, 0.8-1.1 CI 95% and p .901) and for the intention to leave work stressors had a OR of 0.7 with 0.6-0.9 CI 95% p .001 and Energy sources had a OR of 1.3, with 1.2-1.5 CI 95% and p <.001, with the workforce recommendations coming at 1.1 (0.9-1.4, p =.2550  The effects of the work stressors and energy sources from the JDR model have been proven extensively in prior research. The adherence to the workforce recommendations was mediocre, variation was high, and only three recommandations showed (weak but significant) correlation.  Since the workforce recommendations in this study came at no significant OR for the three primary outcomes, as was to be suspected, we decided to put the emphasis in this article on different aspects. | NA |
| Results - I could not find „Supplementary Table 1 – E_table_1.docx“ in my copy of the manuscript. | We thank the reviewer for the feedback on our results section. Supplementary Table 1 – E_table_1.docx has now been renamed S2. Table and was supposerd to be available with the first submission. We will make sure to add it again to the uploaded files as intended. | Page 7 line 203 |
| - I don’t think that the presentation of descriptive results in Table 1 is intuitive. Please consider a complete depiction by separately showing frequencies and percentages for all categorical categories in separate lines, e.g. female/male. | Table 1 has been completed with the comparative groups for readability. In the subscript we added clarification on the used statistical analysis. | Page 7  Table 1 |
| - Which statistical analyses did you use to compute odds rate for results in Table 3? | The OR were calculated using a logistic regression analysis. This was clarified in the text. | Page 8, line 239 |
| Discussion - Please remove references to your tables in the Discussion section. | It has been removed per the suggestion of the reviewer. | Page 11, line 307 |
| References - Please check if the journal recommends to include an English translation in square brackets after foreign manuscript titles, e.g. in citations 1, 15. - Please review all of your references and choose one continuous citation style. | The references were adjusted to the required Vancouver style, as per recommendations by the International Committee of Medical Journal Editors, and PLOS One | Page 13-16 |
| **Editor** |  |  |
| Abstract:  As the aim you state “to evaluate the adherence to the DSEP workforce recommendations, and the association between these recommendations and signs of burnout, work engagement and the intention to leave the profession” | We thank the editor for the valuable commentary given. | NA |
| - Please spell out DSEP (abbreviation cannot be understood) | Line 45 on page 3 now explains the DSEP abbreviation; a small omission on our part. | Page 3  Line 45 |
| - Neither data on the recommendations nor on the associations are presented in the Results section of the abstract. Please add this data. | To improve readability burnout, work engagement and the intention to leave are now referred to as the three main outcomes. The workforce recommendations and their correlation to the three main outcomes were added in the result section of the Abstract. | Page 3  Line 48-52 and  58-63 |
| - I suggest deleting your interpretation/discussion of your findings from the results section of the abstract. Comparison with findings from the literature are usually not made in results section. Also, this relates the impression that you gathered the original data that allowed for such comparisons (e.g. in other health care professions) | We appreciate the advice to remove the interpretation of our findings within the scope of (inter)national literature from the abstract. This removal allowed space to add the results on workforce recommendations mentioned above. | Page 3 line 55-58 |
| Methods in abstract/Methods section:  - Cross-sectional survey-based cohort study please change to cross-sectional study. Cohort is a misleading expression; even if you plan to collect prospective data you are not presenting it at this point. | We have amended the mention of this study being a cohort study. In its intent it is the first wave of a cohort study, but we fully agree that in this single paper it can be misleading. | Page 3, line 48 |
| - Would delete this from the abstract: "this study creates a foundation upon which future research can build and a repeating questionnaire is already planned for end 2025", please present broader implications | The follow-up study is no longer mentioned in the abstract.  We rewrote the conclusion of the abstract with all recommendations from the reviewers in mind. | Page 3, line 72  Line 68-72 |
| - Methods in abstract: how was intention to leave measured? | In the Methods section of the Abstract it was added how the intention to leave was measured: “by three self-developed questions” | Page 3, line 50-51 |
| Methods section in the paper  - The Utrecht Burnout Scale (UBOS): I never heard of the approach to re-name the translation of an original scale (also due to copyright issues). I cannot access the referenced study. Please confirm that the UBOS items are fully identical to the MBI; thus that the UBOS really is close translation and that original MBI items were not removed, significantly revised or that new items were added. Otherwise validity of the UBOS should be documented. | The UBOS is the official Dutch adaptation of the MBI. The Dutch test publisher, Swets & Zeitlinger (now Pearson Test Services), obtained a formal license from the copyright holder, Consulting Psychologists Press (currently Mind Garden, Inc., Palo Alto, CA). The original MBI items were translated into Dutch and subsequently back-translated in accordance with the guidelines prescribed by the copyright holder, ensuring conceptual and linguistic equivalence with the original instrument. A comprehensive Dutch test manual was published in 2001. In our view, this manual provides more extensive documentation than the original MBI manual available at that time. Importantly, more recent editions of the American MBI manual have incorporated empirical data based on the UBOS. Finally, the widespread use of the UBOS is reflected in the scientific literature: a quick search in Google Scholar yields approximately 25,000 hits for “UBOS,” indicating its substantial impact and adoption in both research and practice. | Page 5 line 142 |
| - Work conditions were measured by a self-developed measure. Please show the questionnaire as online supplement to make sure wording of items is transparently shown | If we are not mistaken the entire questionnaire translated to English was provided. We will, with the submission of the reviewed article, make sure it is available to you. | NA |
| - In the methods section you state that you calculated Cronbach’s alpha. Are these reported in the result section for burnout and work engagement? | The Cronbach’s Alphas were not mentioned, but were added. | Page 7, line 206-208 |
| Results: you compared work engagements values to the norm reference values provided by the UWES manual of work engagement scores of Dutch healthcare professionals.  Does this comparison account for gender and age differences between samples? If not: could such differences partly serve as explanations? | This question is absolutely interesting, whether gender and age might serve as an explanation for the differences in work engagement. The entire dataset used in the UWES manual consists of 9.679 participants with a 42.8% male to 57.2% female ratio and a mean age of 38.2.  Our study has a 32.2% male to 67.8% female ratio, and a mean age of 39. Since the entire group seems quite similar in sex and age, we did not further investigate into the demographics of the subgroup. The subgroup scores of 655 (6.8%) Dutch doctors were used as comparison. The demographics within this subgroup are not mentioned in the UWES manual. | NA |
| - In the results section you state that EM physicians score lower on overall work engagement than the reference sample (3.48 vs. 3.74).  This seems inconsistent with the numbers in the table which are 3.48 and 3.10, respectively, which would mean EM physicians score higher. Please check. | How sloppy of us. As a result of combining sentences to reduce word count we made a mistake. Thank you so much for pointing it out: EM physicians scored “higher on” overall work engagement. | Page 7, line 222-223 |
| - Table 3: It seems more intuitive to flip outcomes and exposure of the analyses, as one would implicitly assume that burnout and WE predict the professional outcomes. The logistic regression analyses should be mentioned in the methods section. How did you define exposures and outcomes? | Burnout and work engagement were seen as the exposures and the intention to leave as the outcomes. We flipped table 3. | Page 9  Line 242-243 |
| Discussion  - How do your finding related to WE compare to the literature? | We added a recently published article as reference in which the same UWES-3 tool was used to measure work engagement in 5.023 direct care nurses from 6 European countries. | Page 11, line 304-306 |
| - Limitations: confounding --> no adjustment | We added this limitation in the discussion | Page 11, line 331-332 |
| - Any recommendations for research and practice? | We hoped to have improved our recommendations throughout the text. | Page 11 line 330-333 |
