# Supplementary material for: Workforce guidelines, burnout, work engagement and the intention to leave: A cross-sectional study among Dutch emergency physicians
Source: PLoS One. 2026 Oct 5;21(10):e0337882. doi: 10.1371/journal.pone.0337882 (PMC13637872; doi:10.1371/journal.pone.0337882)
Supplement: Supplementary file 3 [file pone.0337882.s005.docx]

PONE-D-25-60264R1

Workforce guidelines, burnout, work engagement and the intention to leave. A cohort study among Dutch Emergency Physicians.

PLOS ONE

**Response to reviewers**

Dear Editor,

We appreciate the reviewers' valuable comments on our paper ‘Workforce guidelines, burnout, work engagement and the intention to leave. A cross-sectional study among Dutch Emergency Physicians’. We have thoroughly studied the comments and are pleased to provide the following answers and corresponding changes to our paper. We will be happy to provide further elaborations or adjustments to the manuscript if required.

| **Reviewer #1 Comments** | **Revisions (location of revisions in clean manuscript)** | **Page number, line number** |
| --- | --- | --- |
| The authors considerably revised the paper and improved its quality significantly. | We thank the reviewer for the compliments. We appreciate the acknowledgement of the quality of our work. | NA |
| Please carefully proof-read the paper to remove various existing spelling or grammar errors, for instance in the abstract:  - 2 errors: Cross-sectional on line survey-based study among all Dutch EMPs in 2019 --> Cross-sectional online survey based study among all Dutch EMPs in 2019 | ‘Online’ and ‘survey based’ have been corrected. And the paper has been combed through in its entirety for other spelling or grammar errors. | 32  152 |
| - Higher adherence to the workforce recommendations was significantly associated with all three main outcomes, as did five out of twelve separate recommendations” --> as were | Thank you. Changed as suggested. | 43 |
| Methods: The logistic regression analyses should be mentioned in the methods (“analysis and statistics”) section as an analytical strategy (along with exposures and outcomes used) | We have added the logistic regressions to the methods section. | 148-150 |
| Methods: Please present the response options of all the intention to leave variables and explain how you defined “poor outcomes” based on those variables. | Two of the three intention to leave questions were polar questions with only Yes / No options. This was added to the description of the questions in the Methods section. | 132  135-136 |
| Table 3: please check the odds ratios for “considering to quit”. Without clarification readers may interpret you findings in terms of burnout being associated with considerably reduced intentions to quit and work engagement as linked to increased thoughts about quitting. This pattern would be counterintuitive when compared to the literature and when compared to the associations with the other 2 professional outcomes presented in Table 3. Please clarify. | To clarify this, we added “not” to table 3, as in: Not considering to quit. | Table 3 |
| Reviewer #1  The authors have adequately addressed the majority of the reviewers' comments. The manuscript is recommended for acceptance with the following minor observations. | Thank you for your recommendation and your review. |  |
| The 95% CI reported for burnout on "Considering to quit" is 0.1–0.2, which is implausible given an OR of 0.2. This appears to be a typographical error. The lower bound of the CI cannot equal the OR point estimate. This must be verified against the original output and corrected. | This was indeed a typographical error. It has been corrected based on our SPSS Syntax/Output and confirmed by repeated analysis. | Table 3 |
| In the text (Results section) the authors state EMPs scored higher on overall work engagement (3.48 vs. 3.74, p<0.01). The value 3.74 does not match Table 2, which reports the reference group mean as 3.10. The text should read 3.48 vs. 3.10. Although this was flagged by the editor and acknowledged by the authors in the response letter, the error persists in the final clean manuscript and has not been corrected. | Thank you for bringing this to our attention. It has now been corrected. | 185 |
| Reviewer 2 asked why specific demographic variables were chosen for analysis and not others available in the questionnaire (e.g. working hours, weekend work). The authors responded that they were unsure which section of the text the comment referred to, without providing a substantive methodological justification. A brief sentence in the Analysis and Statistics section explaining the rationale for variable selection would strengthen methodological transparency.  Reviewers’ remark referred to:  - Please explain why you choose this specific set of demographic variables and not others from your comprehensive questionnaire, which might have also been suitable with regard to burnout (e.g. working hours (item 13), weekend work (item 28))? | We apologise for still not fully understanding this question.  To clarify, in table 1 we examined the relationship of certain demographics (age, sex, career length, family setting) to our burnout outcome; Table 4 presents the relationship of burnout to the Workforce regulations recommendations (such as working hours, weekend work, shift duration et cetera). These variables (including items 13 and 28) were analysed as a set and were therefore reported separately. |  |
| job securiy" (paragraph 3, Discussion) should read "job security", and "assosiated" should read "associated". Similarly "recommandations" appears in the Strengths and Limitations section instead of "recommendations". These should be corrected before final production. | These and some others have been corrected | 248  252  269  270  275  281  282  293 |
| The alpha of 0.62 for the intention to leave scale is below the conventional threshold of 0.70. While the authors correctly decided to analyze the three items separately, no mention is made in the limitations of the implications this has for the construct validity of this measure. A single sentence acknowledging this as a limitation would be appropriate. | The respondent’s intention to leave was assessed by asking three questions covering what we assumed to be three different aspect of the intention to leave: considering to quit, seeing oneself still in this career in 10 years’ time and choosing the same profession in hindsight. The Cronbach’s alpha value < 0.70 showed that these three questions assessed different aspects of the intention to leave. Consequently, no construct variable was made or used. The three questions were therefore analysed separately throughout the paper. We added a sentence as clarification. | 192-193 |
| These minor corrections notwithstanding, this study provides a valuable baseline for the ongoing longitudinal research and is recommended for publication. | Thank you for your endorsement. |  |
| Reviewer #2: The authors have addressed the reviewer comments in a clear and comprehensible manner, and the revised version of the manuscript is well done. I would recommend a final check of punctuation and spelling, as I noticed a few minor errors. Overall, in my view, the manuscript is ready for publication. | Punctuation and spelling was checked. Thank you for your recommendations. |  |
