# Supplementary material for: Workforce guidelines, burnout, work engagement and the intention to leave: A cross-sectional study among Dutch emergency physicians
Source: PLoS One. 2026 Oct 5;21(10):e0337882. doi: 10.1371/journal.pone.0337882 (PMC13637872; doi:10.1371/journal.pone.0337882)
Supplement: Peer review history

# Peer review: Decision Letter 0

**DOI:** 10.1371/journal.pone.0337882.r001

**Authors:** Adrian Loerbroks

3 Feb 2026

Dear Dr. Boendermaker,

Thank you for submitting your manuscript to PLOS ONE. After careful consideration, we feel that it has merit but does not fully meet PLOS ONE’s publication criteria as it currently stands. Therefore, we invite you to submit a revised version of the manuscript that addresses the points raised during the review process.

Please submit your revised manuscript by Mar 20 2026 11:59PM. If you will need more time than this to complete your revisions, please reply to this message or contact the journal office at plosone@plos.org. When you're ready to submit your revision, log on to https://www.editorialmanager.com/pone/ and select the 'Submissions Needing Revision' folder to locate your manuscript file.

- A letter that responds to each point raised by the academic editor and reviewer(s). You should upload this letter as a separate file labeled 'Response to Reviewers'.
- A marked-up copy of your manuscript that highlights changes made to the original version. You should upload this as a separate file labeled 'Revised Manuscript with Track Changes'.
- An unmarked version of your revised paper without tracked changes. You should upload this as a separate file labeled 'Manuscript'.

If you would like to make changes to your financial disclosure, please include your updated statement in your cover letter. Guidelines for resubmitting your figure files are available below the reviewer comments at the end of this letter.

If applicable, we recommend that you deposit your laboratory protocols in protocols.io to enhance the reproducibility of your results. Protocols.io assigns your protocol its own identifier (DOI) so that it can be cited independently in the future. For instructions see: https://journals.plos.org/plosone/s/submission-guidelines#loc-laboratory-protocols. Additionally, PLOS ONE offers an option for publishing peer-reviewed Lab Protocol articles, which describe protocols hosted on protocols.io. Read more information on sharing protocols at https://plos.org/protocols?utm_medium=editorial-email&utm_source=authorletters&utm_campaign=protocols.

We look forward to receiving your revised manuscript.

Kind regards,

Adrian Loerbroks

Academic Editor

PLOS One

Journal Requirements:

When submitting your revision, we need you to address these additional requirements.

1. Please ensure that your manuscript meets PLOS ONE's style requirements, including those for file naming. The PLOS ONE style templates can be found at

https://journals.plos.org/plosone/s/file?id=wjVg/PLOSOne_formatting_sample_main_body.pdf and

https://journals.plos.org/plosone/s/file?id=ba62/PLOSOne_formatting_sample_title_authors_affiliations.pdf.

2. Thank you for stating the following financial disclosure:

“This study was endorsed by the Dutch Society of Emergency Physicians (DSEP), and received funding from the Dutch Emergency Medicine Research Fund (Stichting Spoedeisende Geneeskunde Onderzoeksfonds. https://sgofonds.nl/). This contribution was only financial in nature and was awarded to main researcher AE Boendermaker, emergency physician and member of the taskforce physician wellness of DSEP.?

Please state what role the funders took in the study.  If the funders had no role, please state: "The funders had no role in study design, data collection and analysis, decision to publish, or preparation of the manuscript."

If this statement is not correct you must amend it as needed.

Please include this amended Role of Funder statement in your cover letter; we will change the online submission form on your behalf.

3. Please be informed that funding information should not appear in the Acknowledgments section or other areas of your manuscript. We will only publish funding information present in the Funding Statement section of the online submission form. Please remove any funding-related text from the manuscript.

4. Please include a caption for figure 1.

5. Please upload a copy of Figure 1, to which you refer in your text on page 5. If the figure is no longer to be included as part of the submission please remove all reference to it within the text.

6. Please include captions for your Supporting Information files at the end of your manuscript, and update any in-text citations to match accordingly. Please see our Supporting Information guidelines for more information: http://journals.plos.org/plosone/s/supporting-information.

7. If the reviewer comments include a recommendation to cite specific previously published works, please review and evaluate these publications to determine whether they are relevant and should be cited. There is no requirement to cite these works unless the editor has indicated otherwise.

[Note: HTML markup is below. Please do not edit.]

Reviewers' comments:

Reviewer's Responses to Questions

**Comments to the Author**

1. Is the manuscript technically sound, and do the data support the conclusions?

Reviewer #1: Yes

Reviewer #2: Yes

**********

2. Has the statistical analysis been performed appropriately and rigorously?

Reviewer #1: Yes

Reviewer #2: Yes

**********

3. Have the authors made all data underlying the findings in their manuscript fully available?

The PLOS Data policy

Reviewer #1: Yes

Reviewer #2: Yes

**********

4. Is the manuscript presented in an intelligible fashion and written in standard English?

Reviewer #1: Yes

Reviewer #2: Yes

**********

Reviewer #1: The manuscript is well conducted, clearly written, and methodologically sound. The use of validated instruments (UBOS and UWES) is appropriate, and the statistical analyses are rigorous. The data support the authors’ conclusions and provide a meaningful contribution to understanding the well-being of emergency physicians in the Netherlands.

However, it is important to emphasize that the statistical correlations identified do not imply causality. They indicate significant associations between workforce recommendations and levels of burnout, work engagement, and intention to leave, but these relationships should not be interpreted as causal. Clarifying this point in the text would help prevent deterministic interpretations.

Additionally, while the questionnaires used are validated and reliable, the burnout measure does not constitute a formal clinical diagnosis. The results should be interpreted as an indicator of burnout tendency or risk, rather than confirmed burnout, which would require assessment by qualified medical or psychological professionals. A short clarification in the methods or discussion would strengthen the accuracy of interpretation.

Finally, as this study was conducted before the COVID-19 pandemic, its findings provide a valuable baseline but may not reflect current realities. Given the substantial changes in emergency care systems worldwide, a follow-up or pre-post study design would be highly relevant to assess potential shifts in burnout and work engagement after the pandemic.

Overall, this is a well-designed and valuable study that addresses an important topic. The manuscript would benefit from the clarifications above, which would enhance its methodological precision and interpretative transparency.

Reviewer #2: Dear authors,

generally, your manuscript is easily legible, well structured and straightforward in its aims, results and discussion. In my view, more elaborate statistical analyses would have been more interesting to the explanation of variance in your outcomes. However, I guess you might have wanted to save these more extensive analyses for the longitudinal data. This manuscript might be suitable for publication after addressing some points of (mostly) clarification and some more substantiation of empirical literature in the Introduction section. I wish you good luck with revising the manuscript.

Abstract

- Please add the following information to the abstract: year of study, mode of survey administration, statistical procedures for data analysis, statistical program for data analysis.

- Please write out abbreviations, e.g. DSEP.

- In your Conclusions section, you repeat the findings from your Results section. I wonder if it would be more interesting to readers to see suggestions how a “decisive action” might look like instead, i.e. ideas on who could address the study results and which strategies might be plausible.

- I would suggest to reconsider pointing out the repeat survey in the abstract. By the time this paper is published, readers might wonder why authors did not prefer to wait for the study data from this successive study to analyze longitudinal data instead of cross-sectional study results.

Background

- The Background section is rather short. Instead of or in addition the findings on the repercussions of burnout for healthcare providers in general, you might refer to empirical findings specifically for EM physicians to establish specific relevance for this professional group. This might also “complete the circle” with regard to the workforce guideline, i.e. why are certain recommendations included in this guideline. The same suggestion applies for work engagement.

- Please add that you measured self-reported adherence and compliance to recommendations. Otherwise, one might confuse it with objective assessments of adherence to recommendations.

Methods

- Please clearly state which part of the study was cross-sectional and which is the cohort study (since a study cannot be cross-sectional and a cohort at the same time). I guess that you analyzed cross-sectional data from one wave of a cohort study. Please explain how many waves were already conducted and how many waves are planned.

- Please shortly explain how you gained access to the email addresses. I guess that you obtained them from the Dutch Society of Emergency Physicians? It would be helpful for international readers to know if every single Dutch emergency physician has to register with the Dutch Society of Emergency Physicians. If not, how many EM physicians are there in the Netherlands in total and how do they compare with those who are not part of the society? Are only emergency physicians working in hospitals eligible to participate or also those working in pre-hospital settings?

- Did you apply any inclusion or exclusion criteria for participants? If yes, please explain.

- Please explain why you choose this specific set of demographic variables and not others from your comprehensive questionnaire, which might have also been suitable with regard to burnout (e.g. working hours (item 13), weekend work (item 28))?

- Please name the amount of items for each burnout domain since your participants answered these items and not the domains per se.

- Why did you use two operationalizations of burnout, i.e. Brenninkmeijer EEplus1 definition and the one provided by the UBOS manual?

- What was the time horizon given in the question on “intention to quit”? Please add this information to the text.

- Please report the Cronbach’s alpha for all of your scales. Please also consider that CA is not a simple measure of “correlation” (page 7) but one of internal consistency.

- I could not find Figure 1 referenced on page 5. Please clarify.

- Please add information on how you addressed missing data in analyses.

- Please explain why you refrained from multivariable logistic regression analyses to include all demographic factors in one analytical model to explain variance in your outcomes?

Results

- I could not find “Supplementary Table 1 – E_table_1.docx“ in my copy of the manuscript.

- I don’t think that the presentation of descriptive results in Table 1 is intuitive. Please consider a complete depiction by separately showing frequencies and percentages for all categorical categories in separate lines, e.g. female/male.

- Which statistical analyses did you use to compute odds rate for results in Table 3?

Discussion

- Please remove references to your tables in the Discussion section.

References

- Please check if the journal recommends to include an English translation in square brackets after foreign manuscript titles, e.g. in citations 1, 15.

- Please review all of your references and choose one continuous citation style.

**********

what does this mean?). If published, this will include your full peer review and any attached files.

If you choose “no”, your identity will remain anonymous but your review may still be made public.

**Do you want your identity to be public for this peer review?**  For information about this choice, including consent withdrawal, please see our Privacy Policy

Reviewer #1: **Yes:** Dr. Giovanni Leonardo Briganti

Reviewer #2: **Yes:** Anna Schneider

**********

[NOTE: If reviewer comments were submitted as an attachment file, they will be attached to this email and accessible via the submission site. Please log into your account, locate the manuscript record, and check for the action link "View Attachments". If this link does not appear, there are no attachment files.]

To ensure your figures meet our technical requirements, please review our figure guidelines: https://journals.plos.org/plosone/s/figures

You may also use PLOS’s free figure tool, NAAS, to help you prepare publication quality figures: https://journals.plos.org/plosone/s/figures#loc-tools-for-figure-preparation.

NAAS will assess whether your figures meet our technical requirements by comparing each figure against our figure specifications.

Comments by the editor:

Abstract:

1) As the aim you state “to evaluate the adherence to the DSEP workforce recommendations, and the association between these recommendations and signs of burnout, work engagement and the intention to leave the profession”

- Please spell out DSEP (abbreviation cannot be understood)

- Neither data on the recommedations nor on the associaitons are presented in the Results section of the abstract. Please add this data.

- I suggest deleting your interpretation/discussion of your findings from the results section of the abstract. Copmarison with findings from the literature are usually not made in results section. Also, this reates the impression that you gathered the original data that allowed for such comparisons (e.g. in other health care prefofessions)

Methods in abstract/Methods seciton:

- Cross-sectional survey-based cohort study please change to cross-sectional study. Cohort is a misleading expression; even if you plan to collect prospective data you are not presenting it at this point.

- Would delete this from the abstract: "this study creates a foundation upon which future research can build and a repeating questionnaire is already planned for end 2025", please present broader implications

;- Methods in abstract: how was intention to leave measured?

“How this study might affect research, practice or policy” --> present broader implications please rather than adverstisement of your follow-up study

Methods section in the paper

- The Utrecht Burnout Scale (UBOS): I never heard of the approach to re-name the translation of an original scale (also due to copyright issues). I cannot access the referenced study. Please confirm that the UBOS items are fully identical to the MBI; thus that the UBOS really is close translation and that original MBI items were not removed, significantly revised or that new items were added. Otherwise valditiy of the UBOS should be documented.

- Work conditions were measured by a self-developed measure. Please show the questionnaire as online supplement to make sure wording of items is transparently shown

- In the methods section you state that you cacluated Cronbach’s alpha. Are these reported in the result section for burnout and work engagement?

- Results: you compared work engagements values to the norm reference values provided by the UWES manual of work engagement scores of Dutch healthcare professionals.  Does this comparison account for gender and age differences between samples? If not: could such differences partly serve as explanations?

- In the results section you state that EM physicans score lower on overall work engagement than the reference sample (3.48 vs. 3.74).  This seems insonsistent with the numbers in the table which are 3.48 and 3.10, respectively, which would mean EM physicans score higher. Please check.

- Table 3: It seems more intuitive to flip outcomes and exposure of the analyses, as one would implicitely assume that burnout and WE predict the professional outcomes. The logistic regression analyses should be metioned in the methods section. How did you define exposures and outcomes?

Discussion

- How do your finding related to WE compare to the literature?

- Limitations: confounding --> no adjustment

- Any recommendations for research and pracitce?

# Author comment: Author response to Decision Letter 1

**DOI:** 10.1371/journal.pone.0337882.r002

20 Apr 2026

The manuscript is well conducted, clearly written, and methodologically sound. The use of validated instruments (UBOS and UWES) is appropriate, and the statistical analyses are rigorous. The data support the authors’ conclusions and provide a meaningful contribution to understanding the well-being of emergency physicians in the Netherlands.

- We thank the reviewer for the compliments. We appreciate the acknowledgement of the quality of our work.

However, it is important to emphasize that the statistical correlations identified do not imply causality. They indicate significant associations between workforce recommendations and levels of burnout, work engagement, and intention to leave, but these relationships should not be interpreted as causal. Clarifying this point in the text would help prevent deterministic interpretations.

- Thank you. We fully agree with the reviewer’s comment. We reviewed the texts to see where we may have caused any confusion. We chose to make a specific statement in the Discussion – Main findings section.

- Page 10, line 285-288

Additionally, while the questionnaires used are validated and reliable, the burnout measure does not constitute a formal clinical diagnosis. The results should be interpreted as an indicator of burnout tendency or risk, rather than confirmed burnout, which would require assessment by qualified medical or psychological professionals. A short clarification in the methods or discussion would strengthen the accuracy of interpretation.

- Valid point. The burnout tool does not measure burnout as a diagnosis on a personal level. We went through the text to ascertain if at any point the text may suggest burnout to be a diagnosis, and we added a clarification in the method section of our paper.

- Page 3 Line 67, Page 4 line 115, Page 5 Line 146, 150-151, 154-155, Page 10, line 289

Finally, as this study was conducted before the COVID-19 pandemic, its findings provide a valuable baseline but may not reflect current realities. Given the substantial changes in emergency care systems worldwide, a follow-up or pre-post study design would be highly relevant to assess potential shifts in burnout and work engagement after the pandemic.

- We have found this a valid point as well and are pleased to announce we are currently collecting data in the follow-up study.

In its first set-up, a follow-up study or longitudinal approach was envisioned, but aimed at the workforce guideline and its adherence. The guideline tested stems from 2015 and a revision was planned for 2020. Hence, the study from 2019 was set to be a baseline and a provider of data for the rewrite. The guideline revision was delayed due to a number of causes, including the COVID-19 pandemic and the recognition of Emergency Medicine as a medical specialty in the Netherlands. The revision has been published in December 2025 and the follow-up study is currently ongoing.

Overall, this is a well-designed and valuable study that addresses an important topic. The manuscript would benefit from the clarifications above, which would enhance its methodological precision and interpretative transparency.

- We thank the reviewer for the opportunity to share our study and address the above mentioned clarifications. We hope to have addressed them to satisfaction.

Reviewer 2

Generally, your manuscript is easily legible, well structured and straightforward in its aims, results and discussion.

- We thank the reviewer for the compliments. We appreciate the acknowledgement of the quality of our work.

In my view, more elaborate statistical analyses would have been more interesting to the explanation of variance in your outcomes. However, I guess you might have wanted to save these more extensive analyses for the longitudinal data.

- Important remark and indeed, most of the focus on variance, and more sturdy predictors will be done in the follow-up study with the longitudinal data.

Abstract

Please add the following information to the abstract: year of study, mode of survey administration, statistical procedures for data analysis, statistical program for data analysis.

- Thank you for these practical tips on improving our abstract.

Abstract – methods line 48 now gives more information on mode of survey, and year. A new sentence is added about the statistical procedures.

- Page 3, line 48. Line 51-53

Please write out abbreviations, e.g. DSEP.

- The text now explains the DSEP abbreviation; a small omission on our part.

- Page 3 line 45

In your Conclusions section, you repeat the findings from your Results section. I wonder if it would be more interesting to readers to see suggestions how a “decisive action” might look like instead, i.e. ideas on who could address the study results and which strategies might be plausible.

- Abstract – conclusion. We removed the repeat of results, to make room for a more detailed recommendation.

- Page 3 Line 68-72

I would suggest to reconsider pointing out the repeat survey in the abstract. By the time this paper is published, readers might wonder why authors did not prefer to wait for the study data from this successive study to analyze longitudinal data instead of cross-sectional study results.

- The mention of the repeat study is removed from the abstract as suggested.

- Page 3

Line 72

The Background section is rather short. Instead of or in addition the findings on the repercussions of burnout for healthcare providers in general, you might refer to empirical findings specifically for EM physicians to establish specific relevance for this professional group. This might also “complete the circle” with regard to the workforce guideline, i.e. why are certain recommendations included in this guideline. The same suggestion applies for work engagement.

- The suggestion the reviewer gives is a valid one. However, there are no findings specific for EMPs on both burnout and work engagement, other than that EMPs overall almost ‘always’ score worst among peers. This is likely mainly due to the 24/7 schedule, in a fast-paced, unpredictable setting, where stressful situations are common. We rewrote the first sentence of the introduction to highlight these differences a bit more.

- Page 4, line 87-88

Please add that you measured self-reported adherence and compliance to recommendations. Otherwise, one might confuse it with objective assessments of adherence to recommendations.

- ‘Self -reported’ was added line 116, page 4

Please clearly state which part of the study was cross-sectional and which is the cohort study (since a study cannot be cross-sectional and a cohort at the same time). I guess that you analyzed cross-sectional data from one wave of a cohort study. Please explain how many waves were already conducted and how many waves are planned.

- This study was the first cross-sectional wave of a cohort study of which the second wave was planned for 5 years later. This has been explained more correctly now on page 5, line 123. Page 1 title page, Page 4 line 115

Please shortly explain how you gained access to the email addresses. I guess that you obtained them from the Dutch Society of Emergency Physicians? It would be helpful for international readers to know if every single Dutch emergency physician has to register with the Dutch Society of Emergency Physicians. If not, how many EM physicians are there in the Netherlands in total and how do they compare with those who are not part of the society? Are only emergency physicians working in hospitals eligible to participate or also those working in pre-hospital settings?

- The Dutch RGS, the registration commission for medical specialists, had 583 registered EMPs at 01-01-2019. At 01-06-2019 DSEP confirmed us to have 526 members. 526/583 equals 90.2% of all Dutch EMPs. Most residencies end in September with approximately 45 new EMPs finishing their training. At 01-01-2020 there were 611 registered EMPs at the RGS, and 603 EMP members of DSEP. 98,7%. We added this in the text.

In the Netherlands all EMP work in EDs as a main part of their job. Prehospital care is not part of our job description.

Both facts are now clarified in the text.

- Page 5, line 124-126

Did you apply any inclusion or exclusion criteria for participants? If yes, please explain.

- The study included all Dutch EMPs registered with the DSEP. We added as per your suggestion a sentence to clarify this in the Analysis and statistics section of the methods.

- Page 6, line 189-194

Please explain why you choose this specific set of demographic variables and not others from your comprehensive questionnaire, which might have also been suitable with regard to burnout (e.g. working hours (item 13), weekend work (item 28))?

- We are unsure which section of text is meant by this comment. We are happy to clarify our choices, but would like to know where specifically the reviewer’s question is applicable to.

Please name the amount of items for each burnout domain since your participants answered these items and not the domains per se.

- The amount of items per burnout domain were added to the text.

- Page 5, line 144-145

Why did you use two operationalizations of burnout, i.e. Brenninkmeijer EEplus1 definition and the one provided by the UBOS manual?

- In literature on burnout the use of the MBI / UBOS is standard. Yet the cut-off for how and when to call cases positive or negative for burn-out symptomatology is varying highly.

Some use only the emotional exhaustion scale (EE), and leave out the other two domains (DP and PA) completely, other use a combination of two out of three domains, and none use the exact same cut-off. In general, studies on burnout report burnout rates going from 20 percent to as high as 90 percent, using the same measurement tool, but different interpretation of the outcomes.

Brenninkmeijers’ proposed EEplus1 (high EE and either high DP ór low PA) and the proposed Burnout Syndrome (both high EE ánd high DP plús low PA) are examples of unifying the way we report on burnout. To determine ‘high EE and DP, and low PA’ the manual suggests to use the 75th percentile of the distribution of the composite variables.

We believe this way we report the most truthful representation of burnout symptoms in our sample.

- Page 5 Line 142-155

What was the time horizon given in the question on “intention to quit”? Please add this information to the text.

- We added this information to the text. In short: having ‘ever’ considered to leave the profession.

- Page 6 Line 169-170

Please report the Cronbach’s alpha for all of your scales. Please also consider that CA is not a simple measure of “correlation” (page 7) but one of internal consistency.

- We thank you for bringing it to our attention that we used the word correlation in the explanation of the Cronbach’s alpha, instead of internal consistency. The CAs were added.

- Page 7, line 206-208

I could not find Figure 1 referenced on page 5. Please clarify.

- The absence of figure 1 you so rightfully point out, is due to one of the final changes we had to make to our article to fit it within the set boundaries for submissions. So sorry, we forgot to remove the reference ‘figure one’.

- Page 6, line 175

Please add information on how you addressed missing data in analyses.

- We added transparency on how we dealt with missing data. The on line questionnaire worked in a way where respondents could only more forward after filling in all previous questions, so missing items was extremely rare.

- Page 6, line 189-194

Please explain why you refrained from multivariable logistic regression analyses to include all demographic factors in one analytical model to explain variance in your outcomes?

- A multivariate logistic regression analysis was performed correcting for age, sex, career length, and the most common work stressors and energy sources. These work stressors and energy sources were derived from the job demands and resources model (Schaufeli W, Bakker A, 2004). This model uses work pressure, role conflict and emotional burden as three well established job demands, and possibilities for personal growth, work fit, autonomy, peer support, team atmosphere and team effectiveness as six well known job resources.

For burnout as expected energy sources ( OR 0.8, with 0.7-0.9 CI 95% and p .003) and work stressors (OR 1.9 and 1.4-2.5 CI 95% at p <.0001) proved to be strong predictors. We tested the composite variable of the 12 workforce factors, which showed a calculated OR of 0.9 with 0.9-1.1 CI 95% for burnout.

Only the energy sources were of predictive value for work engagement (workforce came at OR 0.9, 0.8-1.1 CI 95% and p .901) and for the intention to leave work stressors had a OR of 0.7 with 0.6-0.9 CI 95% p .001 and Energy sources had a OR of 1.3, with 1.2-1.5 CI 95% and p <.001, with the workforce recommendations coming at 1.1 (0.9-1.4, p =.2550

The effects of the work stressors and energy sources from the JDR model have been proven extensively in prior research. The adherence to the workforce recommendations was mediocre, variation was high, and only three recommandations showed (weak but significant) correlation.

Since the workforce recommendations in this study came at no significant OR for the three primary outcomes, as was to be suspected, we decided to put the emphasis in this article on different aspects.

Results - I could not find „Supplementary Table 1 – E_table_1.docx“ in my copy of the manuscript.

- We thank the reviewer for the feedback on our results section. Supplementary Table 1 – E_table_1.docx has now been renamed S2. Table and was supposerd to be available with the first submission. We will make sure to add it again to the uploaded files as intended.

I don’t think that the presentation of descriptive results in Table 1 is intuitive. Please consider a complete depiction by separately showing frequencies and percentages for all categorical categories in separate lines, e.g. female/male.

- Table 1 has been completed with the comparative groups for readability. In the subscript we added clarification on the used statistical analysis.

- Page 7

Which statistical analyses did you use to compute odds rate for results in Table 3?

- The OR were calculated using a logistic regression analysis. This was clarified in the text.

- Page 8, line 239

Discussion - Please remove references to your tables in the Discussion section.

- It has been removed per the suggestion of the reviewer.

- Page 11, line 307

References - Please check if the journal recommends to include an English translation in square brackets after foreign manuscript titles, e.g. in citations 1, 15. Please review all of your references and choose one continuous citation style.

- The references were adjusted to the required Vancouver style, as per recommendations by the International Committee of Medical Journal Editors, and PLOS One

Editor

Abstract:

As the aim you state “to evaluate the adherence to the DSEP workforce recommendations, and the association between these recommendations and signs of burnout, work engagement and the intention to leave the profession”

- We thank the editor for the valuable commentary given.

Please spell out DSEP (abbreviation cannot be understood)

- Line 45 on page 3 now explains the DSEP abbreviation; a small omission on our part.

Neither data on the recommendations nor on the associations are presented in the Results section of the abstract. Please add this data.

- To improve readability burnout, work engagement and the intention to leave are now referred to as the three main outcomes. The workforce recommendations and their correlation to the three main outcomes were added in the result section of the Abstract.

- Page 3, line 48-52, 58-63

I suggest deleting your interpretation/discussion of your findings from the results section of the abstract. Comparison with findings from the literature are usually not made in results section. Also, this relates the impression that you gathered the original data that allowed for such comparisons (e.g. in other health care professions)

- We appreciate the advice to remove the interpretation of our findings within the scope of (inter)national literature from the abstract. This removal allowed space to add the results on workforce recommendations mentioned above.

- Page 3, line 55-58

Methods in abstract/Methods section: Cross-sectional survey-based cohort study please change to cross-sectional study. Cohort is a misleading expression; even if you plan to collect prospective data you are not presenting it at this point.

- We have amended the mention of this study being a cohort study. In its intent it is the first wave of a cohort study, but we fully agree that in this single paper it can be misleading.

- Page 3, line 48

Would delete this from the abstract: "this study creates a foundation upon which future research can build and a repeating questionnaire is already planned for end 2025", please present broader implications

- The follow-up study is no longer mentioned in the abstract. We rewrote the conclusion of the abstract with all recommendations from the reviewers in mind.

- Page 3, line 72, Line 68-72

Methods in abstract: how was intention to leave measured?

- In the Methods section of the Abstract it was added how the intention to leave was measured: “by three self-developed questions”

- Page 3, line 50-51

Methods section in the paper: The Utrecht Burnout Scale (UBOS): I never heard of the approach to re-name the translation of an original scale (also due to copyright issues). I cannot access the referenced study. Please confirm that the UBOS items are fully identical to the MBI; thus that the UBOS really is close translation and that original MBI items were not removed, significantly revised or that new items were added. Otherwise validity of the UBOS should be documented.

- The UBOS is the official Dutch adaptation of the MBI. The Dutch test publisher, Swets & Zeitlinger (now Pearson Test Services), obtained a formal license from the copyright holder, Consulting Psychologists Press (currently Mind Garden, Inc., Palo Alto, CA). The original MBI items were translated into Dutch and subsequently back-translated in accordance with the guidelines prescribed by the copyright holder, ensuring conceptual and linguistic equivalence with the original instrument. A comprehensive Dutch test manual was published in 2001. In our view, this manual provides more extensive documentation than the original MBI manual available at that time. Importantly, more recent editions of the American MBI manual have incorporated empirical data based on the UBOS. Finally, the widespread use of the UBOS is reflected in the scientific literature: a quick search in Google Scholar yields approximately 25,000 hits for “UBOS,” indicating its substantial impact and adoption in both research and practice.

- Page 5 line 142

Work conditions were measured by a self-developed measure. Please show the questionnaire as online supplement to make sure wording of items is transparently shown

- If we are not mistaken the entire questionnaire translated to English was provided. We will, with the submission of the reviewed article, make sure it is available to you.

In the methods section you state that you calculated Cronbach’s alpha. Are these reported in the result section for burnout and work engagement?

- The Cronbach’s Alphas were not mentioned, but were added.

- Page 7, line 206-208

Results: you compared work engagements values to the norm reference values provided by the UWES manual of work engagement scores of Dutch healthcare professionals. Does this comparison account for gender and age differences between samples? If not: could such differences partly serve as explanations?

- This question is absolutely interesting, whether gender and age might serve as an explanation for the differences in work engagement. The entire dataset used in the UWES manual consists of 9.679 participants with a 42.8% male to 57.2% female ratio and a mean age of 38.2.

Our study has a 32.2% male to 67.8% female ratio, and a mean age of 39. Since the entire group seems quite similar in sex and age, we did not further investigate into the demographics of the subgroup. The subgroup scores of 655 (6.8%) Dutch doctors were used as comparison. The demographics within this subgroup are not mentioned in the UWES manual.

In the results section you state that EM physicians score lower on overall work engagement than the reference sample (3.48 vs. 3.74). This seems inconsistent with the numbers in the table which are 3.48 and 3.10, respectively, which would mean EM physicians score higher. Please check.

- How sloppy of us. As a result of combining sentences to reduce word count we made a mistake. Thank you so much for pointing it out: EM physicians scored “higher on” overall work engagement.

- Page 7, line 222-223

Table 3: It seems more intuitive to flip outcomes and exposure of the analyses, as one would implicitly assume that burnout and WE predict the professional outcomes. The logistic regression analyses should be mentioned in the methods section. How did you define exposures and outcomes?

- Burnout and work engagement were seen as the exposures and the intention to leave as the outcomes. We flipped table 3. Page 9

Discussion - How do your finding related to WE compare to the literature?

- We added a recently published article as reference in which the same UWES-3 tool was used to measure work engagement in 5.023 direct care nurses from 6 European countries.

- Page 11, line 304-306

Limitations: confounding --> no adjustment

- We added this limitation in the discussion

- Page 11, line 331-332

Any recommendations for research and practice?

- We hope to have improved our recommendations throughout the text.

- Page 11 line 330-333

We thank the editor and reviewers for their thorough work and helpfull recommendations. We will be pleased to further elaborate, or adjust the manuscript if any of the comments were not sufficiently implemented or explained.

Attachment Submitted filename: Response to reviewers PONE-D-25-60264.docx

# Peer review: Decision Letter 1

**DOI:** 10.1371/journal.pone.0337882.r003

**Authors:** Adrian Loerbroks

12 Jun 2026

Dear Dr. Boendermaker,

Thank you for submitting your manuscript to PLOS ONE. After careful consideration, we feel that it has merit but does not fully meet PLOS ONE’s publication criteria as it currently stands. Therefore, we invite you to submit a revised version of the manuscript that addresses the points raised during the review process.

Please submit your revised manuscript by Jul 27 2026 11:59PM. If you will need more time than this to complete your revisions, please reply to this message or contact the journal office at plosone@plos.org. When you're ready to submit your revision, log on to https://www.editorialmanager.com/pone/ and select the 'Submissions Needing Revision' folder to locate your manuscript file.

- A letter that responds to each point raised by the academic editor and reviewer(s). You should upload this letter as a separate file labeled 'Response to Reviewers'.
- A marked-up copy of your manuscript that highlights changes made to the original version. You should upload this as a separate file labeled 'Revised Manuscript with Track Changes'.
- An unmarked version of your revised paper without tracked changes. You should upload this as a separate file labeled 'Manuscript'.

If you would like to make changes to your financial disclosure, please include your updated statement in your cover letter. Guidelines for resubmitting your figure files are available below the reviewer comments at the end of this letter.

If applicable, we recommend that you deposit your laboratory protocols in protocols.io to enhance the reproducibility of your results. Protocols.io assigns your protocol its own identifier (DOI) so that it can be cited independently in the future. For instructions see: https://journals.plos.org/plosone/s/submission-guidelines#loc-laboratory-protocols. Additionally, PLOS ONE offers an option for publishing peer-reviewed Lab Protocol articles, which describe protocols hosted on protocols.io. Read more information on sharing protocols at https://plos.org/protocols?utm_medium=editorial-email&utm_source=authorletters&utm_campaign=protocols.

As the corresponding author, your ORCID iD is verified in the submission system and will appear in the published article. PLOS supports the use of ORCID, and we encourage all coauthors to register for an ORCID iD and use it as well. Please encourage your coauthors to verify their ORCID iD within the submission system before final acceptance, as unverified ORCID iDs will not appear in the published article. *Only*  the individual author can complete the verification step; PLOS staff *cannot*  verify ORCID iDs on behalf of authors.

We look forward to receiving your revised manuscript.

Kind regards,

Adrian Loerbroks

Academic Editor

PLOS One

Journal Requirements:

1. If the reviewer comments include a recommendation to cite specific previously published works, please review and evaluate these publications to determine whether they are relevant and should be cited. There is no requirement to cite these works unless the editor has indicated otherwise.

2. Please review your reference list to ensure that it is complete and correct. If you have cited papers that have been retracted, please include the rationale for doing so in the manuscript text, or remove these references and replace them with relevant current references. Any changes to the reference list should be mentioned in the rebuttal letter that accompanies your revised manuscript. If you need to cite a retracted article, indicate the article’s retracted status in the References list and also include a citation and full reference for the retraction notice.

Additional Editor Comments:

The authors considerably revised the paper and improved its quality significantly. I have only a few final comments/requests:

1. Please carefully proof-read the paper to remove various existing spelling or grammar errors, for instance in the abstract:

- 2 errors: Cross-sectional on line survey-based study among all Dutch EMPs in 2019 --> Cross-sectional online survey based study among all Dutch EMPs in 2019

- Higher adherence to the workforce recommendations was significantly associated with all three main outcomes, as did five out of twelve separate recommendations” --> as were

2. Methods: The logistic regression analyses should be mentioned in the methods (“analysis and statistics”) section as an analytical strategy (along with exposures and outcomes used)

3. Methods: Please present the response options of all the intention to leave variables and explain how you defined “poor outcomes” based on those variables.

4. Table 3: please check the odds ratios for “considering to quit”. Without clarification readers may interpret you findings in terms of burnout being associated with considerably reduced intentions to quit and work engagement as linked to increased thoughts about quitting. This pattern would be counterintuitive when compared to the literature and when compared to the associations with the other 2 professional outcomes presented in Table 3. Please clarify.

[Note: HTML markup is below. Please do not edit.]

Reviewers' comments:

Reviewer's Responses to Questions

**Comments to the Author**

Reviewer #1: (No Response)

Reviewer #2: All comments have been addressed

**********

2. Is the manuscript technically sound, and do the data support the conclusions?

Reviewer #1: Yes

Reviewer #2: Yes

**********

3. Has the statistical analysis been performed appropriately and rigorously?

Reviewer #1: Yes

Reviewer #2: Yes

**********

4. Have the authors made all data underlying the findings in their manuscript fully available?

The PLOS Data policy

Reviewer #1: Yes

Reviewer #2: No

**********

5. Is the manuscript presented in an intelligible fashion and written in standard English?

Reviewer #1: Yes

Reviewer #2: Yes

**********

Reviewer #1: The authors have adequately addressed the majority of the reviewers' comments. The manuscript is recommended for acceptance with the following minor observations.

1. The 95% CI reported for burnout on "Considering to quit" is 0.1–0.2, which is implausible given an OR of 0.2. This appears to be a typographical error. The lower bound of the CI cannot equal the OR point estimate. This must be verified against the original output and corrected.

2. In the text (Results section) the authors state EMPs scored higher on overall work engagement (3.48 vs. 3.74, p<0.01). The value 3.74 does not match Table 2, which reports the reference group mean as 3.10. The text should read 3.48 vs. 3.10. Although this was flagged by the editor and acknowledged by the authors in the response letter, the error persists in the final clean manuscript and has not been corrected.

3. Reviewer 2 asked why specific demographic variables were chosen for analysis and not others available in the questionnaire (e.g. working hours, weekend work). The authors responded that they were unsure which section of the text the comment referred to, without providing a substantive methodological justification. A brief sentence in the Analysis and Statistics section explaining the rationale for variable selection would strengthen methodological transparency.

4. "job securiy" (paragraph 3, Discussion) should read "job security", and "assosiated" should read "associated". Similarly "recommandations" appears in the Strengths and Limitations section instead of "recommendations". These should be corrected before final production.

5. The alpha of 0.62 for the intention to leave scale is below the conventional threshold of 0.70. While the authors correctly decided to analyse the three items separately, no mention is made in the limitations of the implications this has for the construct validity of this measure. A single sentence acknowledging this as a limitation would be appropriate.

These minor corrections notwithstanding, this study provides a valuable baseline for the ongoing longitudinal research and is recommended for publication.

Reviewer #2: The authors have addressed the reviewer comments in a clear and comprehensible manner, and the revised version of the manuscript is well done. I would recommend a final check of punctuation and spelling, as I noticed a few minor errors. Overall, in my view, the manuscript is ready for publication.

**********

what does this mean?). If published, this will include your full peer review and any attached files.

If you choose “no”, your identity will remain anonymous but your review may still be made public.

**Do you want your identity to be public for this peer review?**  For information about this choice, including consent withdrawal, please see our Privacy Policy

Reviewer #1: **Yes:** Dr. Giovanni Leonardo Briganti

Reviewer #2: **Yes:** Anna Schneider

**********

[NOTE: If reviewer comments were submitted as an attachment file, they will be attached to this email and accessible via the submission site. Please log into your account, locate the manuscript record, and check for the action link "View Attachments". If this link does not appear, there are no attachment files.]

To ensure your figures meet our technical requirements, please review our figure guidelines: https://journals.plos.org/plosone/s/figures

You may also use PLOS’s free figure tool, NAAS, to help you prepare publication quality figures: https://journals.plos.org/plosone/s/figures#loc-tools-for-figure-preparation.

NAAS will assess whether your figures meet our technical requirements by comparing each figure against our figure specifications.

# Author comment: Author response to Decision Letter 2

**DOI:** 10.1371/journal.pone.0337882.r004

25 Jul 2026

Dear Editor,

We appreciate the reviewers' valuable comments on our paper ‘Workforce guidelines, burnout, work engagement and the intention to leave. A cross-sectional study among Dutch Emergency Physicians’. We have thoroughly studied the comments and are pleased to provide the following answers and corresponding changes to our paper. We will be happy to provide further elaborations or adjustments to the manuscript if required.

Reviewer #1

- The authors considerably revised the paper and improved its quality significantly.

We thank the reviewer for the compliments. We appreciate the acknowledgement of the quality of our work.

- Please carefully proof-read the paper to remove various existing spelling or grammar errors, for instance in the abstract:

- 2 errors: Cross-sectional on line survey-based study among all Dutch EMPs in 2019 --> Cross-sectional online survey based study among all Dutch EMPs in 2019

‘Online’ and ‘survey based’ have been corrected. And the paper has been combed through in its entirety for other spelling or grammar errors. Lines 32

152

- Higher adherence to the workforce recommendations was significantly associated with all three main outcomes, as did five out of twelve separate recommendations” --> as were

Thank you. Changed as suggested. Line 43

- Methods: The logistic regression analyses should be mentioned in the methods (“analysis and statistics”) section as an analytical strategy (along with exposures and outcomes used)

We have added the logistic regressions to the methods section. Lines 148-150

- Methods: Please present the response options of all the intention to leave variables and explain how you defined “poor outcomes” based on those variables.

Two of the three intention to leave questions were polar questions with only Yes / No options. This was added to the description of the questions in the Methods section. Lines 132, 135-136

- Table 3: please check the odds ratios for “considering to quit”. Without clarification readers may interpret you findings in terms of burnout being associated with considerably reduced intentions to quit and work engagement as linked to increased thoughts about quitting. This pattern would be counterintuitive when compared to the literature and when compared to the associations with the other 2 professional outcomes presented in Table 3. Please clarify.

To clarify this, we added “not” to table 3, as in: Not considering to quit. Table 3

Reviewer #1

- The authors have adequately addressed the majority of the reviewers' comments. The manuscript is recommended for acceptance with the following minor observations.

Thank you for your recommendation and your review.

- The 95% CI reported for burnout on "Considering to quit" is 0.1–0.2, which is implausible given an OR of 0.2. This appears to be a typographical error. The lower bound of the CI cannot equal the OR point estimate. This must be verified against the original output and corrected.

This was indeed a typographical error. It has been corrected based on our SPSS Syntax/Output and confirmed by repeated analysis. Table 3

- In the text (Results section) the authors state EMPs scored higher on overall work engagement (3.48 vs. 3.74, p<0.01). The value 3.74 does not match Table 2, which reports the reference group mean as 3.10. The text should read 3.48 vs. 3.10. Although this was flagged by the editor and acknowledged by the authors in the response letter, the error persists in the final clean manuscript and has not been corrected.

Thank you for bringing this to our attention. It has now been corrected. Line 185

- Reviewer 2 asked why specific demographic variables were chosen for analysis and not others available in the questionnaire (e.g. working hours, weekend work). The authors responded that they were unsure which section of the text the comment referred to, without providing a substantive methodological justification. A brief sentence in the Analysis and Statistics section explaining the rationale for variable selection would strengthen methodological transparency.

Reviewers’ remark referred to:

- Please explain why you choose this specific set of demographic variables and not others from your comprehensive questionnaire, which might have also been suitable with regard to burnout (e.g. working hours (item 13), weekend work (item 28))?

We apologise for still not fully understanding this question.

To clarify, in table 1 we examined the relationship of certain demographics (age, sex, career length, family setting) to our burnout outcome; Table 4 presents the relationship of burnout to the Workforce regulations recommendations (such as working hours, weekend work, shift duration et cetera). These variables (including items 13 and 28) were analysed as a set and were therefore reported separately.

- job securiy" (paragraph 3, Discussion) should read "job security", and "assosiated" should read "associated". Similarly "recommandations" appears in the Strengths and Limitations section instead of "recommendations". These should be corrected before final production.

These and some others have been corrected Lines 248, 252, 269, 270, 275, 281, 282, 293

- The alpha of 0.62 for the intention to leave scale is below the conventional threshold of 0.70. While the authors correctly decided to analyze the three items separately, no mention is made in the limitations of the implications this has for the construct validity of this measure. A single sentence acknowledging this as a limitation would be appropriate.

The respondent’s intention to leave was assessed by asking three questions covering what we assumed to be three different aspect of the intention to leave: considering to quit, seeing oneself still in this career in 10 years’ time and choosing the same profession in hindsight. The Cronbach’s alpha value < 0.70 showed that these three questions assessed different aspects of the intention to leave. Consequently, no construct variable was made or used. The three questions were therefore analysed separately throughout the paper. We added a sentence as clarification. Lines 192-193

- These minor corrections notwithstanding, this study provides a valuable baseline for the ongoing longitudinal research and is recommended for publication.

Thank you for your endorsement.

- Reviewer #2: The authors have addressed the reviewer comments in a clear and comprehensible manner, and the revised version of the manuscript is well done. I would recommend a final check of punctuation and spelling, as I noticed a few minor errors. Overall, in my view, the manuscript is ready for publication.

Punctuation and spelling was checked. Thank you for your recommendations.

Attachment Submitted filename: Response to reviewers PONE-D-25-60264R2.docx

# Editor report: Decision Letter 2

**DOI:** 10.1371/journal.pone.0337882.r005

**Authors:** Adrian Loerbroks

30 Jul 2026

<p>Workforce guidelines, burnout, work engagement and the intention to leave. A cross sectional study among Dutch Emergency Physicians.

PONE-D-25-60264R2

Dear Dr. Boendermaker,

We’re pleased to inform you that your manuscript has been judged scientifically suitable for publication and will be formally accepted for publication once it meets all outstanding technical requirements.

Within one week, you’ll receive an e-mail detailing the required amendments. When these have been addressed, you’ll receive a formal acceptance letter and your manuscript will be scheduled for publication.

An invoice will be generated when your article is formally accepted. Please note, if your institution has a publishing partnership with PLOS and your article meets the relevant criteria, all or part of your publication costs will be covered. Please make sure your user information is up-to-date by logging into Editorial Manager at Editorial Manager® and clicking the ‘Update My Information' link at the top of the page. For questions related to billing, please contact billing support.

If your institution or institutions have a press office, please notify them about your upcoming paper to help maximize its impact. If they’ll be preparing press materials, please inform our press team as soon as possible -- no later than 48 hours after receiving the formal acceptance. Your manuscript will remain under strict press embargo until 2 pm Eastern Time on the date of publication. For more information, please contact onepress@plos.org.

Kind regards,

Adrian Loerbroks

Academic Editor

PLOS One

Additional Editor Comments (optional):

Reviewers' comments:

# Editor report: Acceptance letter

**DOI:** 10.1371/journal.pone.0337882.r006

**Authors:** Adrian Loerbroks

PONE-D-25-60264R2

PLOS One

Dear Dr. Boendermaker,

I'm pleased to inform you that your manuscript has been deemed suitable for publication in PLOS One. Congratulations! Your manuscript is now being handed over to our production team.

At this stage, our production department will prepare your paper for publication. This includes ensuring the following:

* All references, tables, and figures are properly cited

* All relevant supporting information is included in the manuscript submission,

* There are no issues that prevent the paper from being properly typeset

You will receive further instructions from the production team, including instructions on how to review your proof when it is ready. Please keep in mind that we are working through a large volume of accepted articles, so please give us a few days to review your paper and let you know the next and final steps.

Lastly, if your institution or institutions have a press office, please let them know about your upcoming paper now to help maximize its impact. If they'll be preparing press materials, please inform our press team within the next 48 hours. Your manuscript will remain under strict press embargo until 2 pm Eastern Time on the date of publication. For more information, please contact onepress@plos.org.

You will receive an invoice from PLOS for your publication fee after your manuscript has reached the completed accept phase. If you receive an email requesting payment before acceptance or for any other service, this may be a phishing scheme. Learn how to identify phishing emails and protect your accounts at https://explore.plos.org/phishing.

If we can help with anything else, please email us at customercare@plos.org.

Thank you for submitting your work to PLOS One and supporting open access.

Kind regards,

PLOS One Editorial Office Staff

on behalf of

Dr. Adrian Loerbroks

Academic Editor

PLOS One
